# Cheminformatics-based identification of phosphorylated RET tyrosine kinase inhibitors for human cancer

**DOI:** 10.3389/fchem.2024.1407331

**Published:** 2024-07-17

**Authors:** Md. Enamul Kabir Talukder, Md. Aktaruzzaman, Noimul Hasan Siddiquee, Sabrina Islam, Tanveer A. Wani, Hamad M. Alkahtani, Seema Zargar, Md. Obayed Raihan, Md. Mashiar Rahman, Sushil Pokhrel, Foysal Ahammad

**Affiliations:** ^1^ Laboratory of Computational Biology, Biological Solution Centre, Jashore, Bangladesh; ^2^ Department of Genetic Engineering and Biotechnology, Jashore University of Science and Technology, Jashore, Bangladesh; ^3^ Department of Pharmacy, Jashore University of Science and Technology, Jashore, Bangladesh; ^4^ Department of Microbiology, Noakhali Science and Technology University, Noakhali, Bangladesh; ^5^ Biological Sciences Department, Florida Atlantic University, Boca Raton, FL, United States; ^6^ Department of Pharmaceutical Chemistry, College of Pharmacy, King Saud University, Riyadh, Saudi Arabia; ^7^ Department of Biochemistry, College of Science, King Saud University, Riyadh, Saudi Arabia; ^8^ Department of Pharmaceutical Sciences, College of Pharmacy, Chicago State University, Chicago, IL, United States; ^9^ Department of Biomedical Engineering, State University of New York at Binghamton SUNY, Binghamton, NY, United States; ^10^ Division of Biological and Biomedical Sciences (BBS), College of Health and Life Sciences (CHLS), Hamad Bin Khalifa University (HBKU), Doha, Qatar

**Keywords:** phosphorylated rearranged during transfection tyrosine kinase, cancer, high-throughput virtual screening, extra precision docking, docking validation, phase database, molecular mechanics with generalized Born surface area, molecular dynamics simulation

## Abstract

**Background:**

Rearranged during transfection (RET), an oncogenic protein, is associated with various cancers, including non-small-cell lung cancer (NSCLC), papillary thyroid cancer (PTC), pancreatic cancer, medullary thyroid cancer (MTC), breast cancer, and colorectal cancer. Dysregulation of RET contributes to cancer development, highlighting the importance of identifying lead compounds targeting this protein due to its pivotal role in cancer progression. Therefore, this study aims to discover effective lead compounds targeting RET across different cancer types and evaluate their potential to inhibit cancer progression.

**Methods:**

This study used a range of computational techniques, including Phase database creation, high-throughput virtual screening (HTVS), molecular docking, molecular mechanics with generalized Born surface area (MM-GBSA) solvation, assessment of pharmacokinetic (PK) properties, and molecular dynamics (MD) simulations, to identify potential lead compounds targeting RET.

**Results:**

Initially, a high-throughput virtual screening of the ZINC database identified 2,550 compounds from a pool of 170,269. Subsequent molecular docking studies revealed 10 compounds with promising negative binding scores ranging from −8.458 to −7.791 kcal/mol. MM-GBSA analysis further confirmed the potential of four compounds to exhibit negative binding scores. MD simulations demonstrated the stability of CID 95842900, CID 137030374, CID 124958150, and CID 110126793 with the target receptors.

**Conclusion:**

These findings suggest that these selected four compounds have the potential to inhibit phosphorylated RET (pRET) tyrosine kinase activity and may represent promising candidates for the treatment of various cancers.

## 1 Introduction

Cancer remains a pressing global health concern, with its incidence increasing due to various lifestyle factors (Mathi et al., 2014). The World Health Organization (WHO) reported approximately 9.6 million cancer deaths in 2018, (Ravi Kumar et al., 2020), with projections indicating an increase to 11.5 million by 2030 (Gurung et al., 2021). An important characteristic of cancer is aberrant cell proliferation, which is caused by dysregulation of the cell cycle. There are several factors that govern the cell cycle in order to ensure that the cells divide regularly and in a programmed manner (Asadi-Samani et al., 2022). Key players in this process are cell surface receptors, particularly receptor tyrosine kinases (RTKs), which control vital signaling pathways involved in various cellular processes, including cell growth, differentiation, and survival (O’Leary et al., 2019). Dysregulation of RTKs, notably the rearranged during transfection (RET) kinase, contributes to tumorigenesis in various cancers.

**FIGURE 1:**
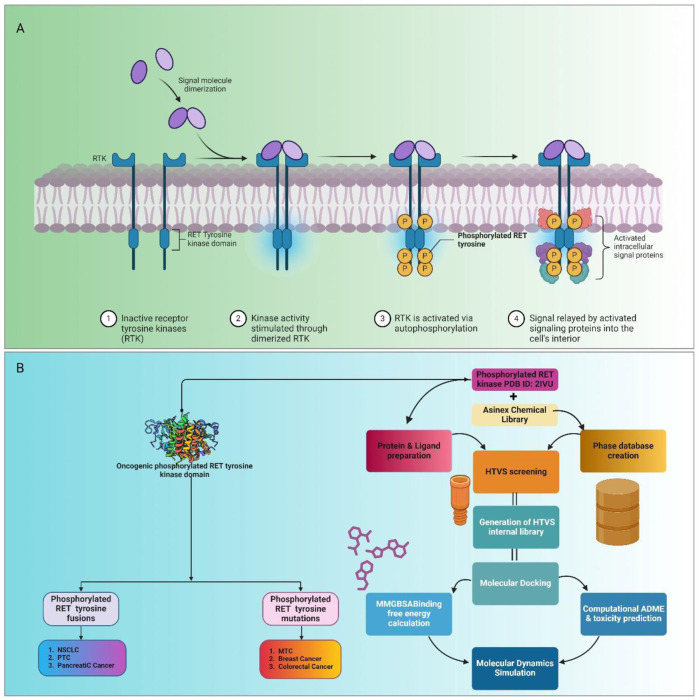
Mechanism of action and inhibition of phosphorylated RET tyrosine kinase activity by potential compounds. **(A)** Role of phosphorylated RET tyrosine kinase in cancer progression. Phosphorylated RET, through mutations and aberrant activation, promotes uncontrolled cell growth and inhibits apoptosis, leading to tumor development and metastasis. RET fusion and mutation are implicated in various cancers, including NSCLC, papillary thyroid cancer (PTC), and medullary thyroid cancer (MTC). **(B)** Study’s efforts to identify potential compounds that can effectively inhibit phosphorylated RET tyrosine kinase, offering an alternative to the existing inhibitor, vandetanib.

RTKs are multifaceted proteins crucial for modulating cell activities like growth, differentiation, and survival (Mahato and Sidorova, 2020; Lemmon and Schlessinger, 2010). Mutation-induced dysregulation of RTKs promotes uncontrolled cell growth and prevents cell death, promoting tumor progression and metastasis (Zhang and Li, 2023). The aberrant activation of RTKs, particularly by RET fusion and mutation, is implicated in numerous cancers, spanning non-small-cell lung cancer (NSCLC) to breast cancer (Bhujbal et al., 2021). The RET gene encodes a receptor tyrosine kinase that, upon binding with its ligands, undergoes dimerization and auto-phosphorylation on specific tyrosine residues within its intracellular domain (Figure 1A). This phosphorylation triggers the activation of downstream signaling pathways, including the RAS/MAPK, PI3K/AKT, and JAK/STAT pathways, which are essential for regulating cell growth, differentiation, and survival (Ardito et al., 2017). Mutations and gene fusions involving RET lead to its constitutive activation, meaning that the kinase remains continuously active without the need for ligand binding (Liu et al., 2020). This aberrant activation results in uncontrolled cellular proliferation, resistance to apoptosis (programmed cell death), and increased cell migration, all of which contribute to tumorigenesis and cancer metastasis (Neophytou et al., 2021). Consequently, therapeutic strategies targeting RTKs, specifically RET, have gained traction in anticancer research (Hojjat-Farsangi, 2014). By inhibiting RET signaling, we can disrupt the abnormal cellular processes it initiates, offering a promising treatment option. Targeting RET has shown significant success, particularly in cancers characterized by specific genetic alterations involving the RET kinase (Parate et al., 2022b).

Over the past two decades, small molecule-based tyrosine kinase inhibitors (TKIs) have emerged as promising therapies for various cancers (Saraon et al., 2021). First-generation RET inhibitors like vandetanib and their second-generation counterparts, such as selpercatinib, have shown efficacy in inhibiting RET kinase activity. However, adverse effects like hypertension and diarrhea pose significant clinical challenges (Sharma et al., 2016). The search for novel RET inhibitors with improved safety profiles remains crucial to optimize therapeutic outcomes and mitigate side effects (Puji et al., 2021)^.^

To address these challenges, computational approaches have innovated drug discovery efforts (Daoui et al., 2023). Employing techniques like structure-based drug design, molecular docking, molecular dynamics (MD) simulation, molecular mechanics with generalized Born surface area (MM-GBSA), absorption, distribution, metabolism, excretion, and toxicity (ADMET), and others, researchers have accelerated the identification of novel RET inhibitors with enhanced potency and selectivity. Therefore, this study aimed to provide a comprehensive overview of RET kinase inhibitors, encompassing their mechanisms, efficacy, and safety profiles. Using computational approaches, including high-throughput virtual screening (HTVS), molecular docking, and MD simulation, we identified lead compounds with promising therapeutic potential (Figure 1B). Our findings underscore the pivotal role of computational drug discovery in advancing precision oncology, paving the way for tailored therapeutic interventions in RET-driven malignancies. Cancer remains a significant challenge, necessitating innovative therapeutic strategies (Sabe et al., 2021)^.^ By understanding the landscape of RET kinase inhibitors and using computational methodologies, we aim to translate these insights into clinical practice, ultimately enhancing patient outcomes and revolutionizing cancer treatment paradigms.

## 2 Materials and methods

### 2.1 Protein retrieval and preparation

The crystal structure of the phosphorylated RET (pRET) tyrosine kinase region (PDB ID: 2IVU) complexed with vandetanib (ZD6474), a native inhibitor, was obtained from the Protein Data Bank (https://www.rcsb.org/) (Knowles et al., 2006). Due to the presence of heteroatoms, water molecules, and solvents in the structure, it was unsuitable for molecular docking and further studies. Hence, the Schrödinger Protein Preparation Wizard was employed to optimize the protein structure. This involved the addition of hydrogen atoms and missing residues, adjustment of formal charges on hetero groups, establishment of tautomeric and ionization states at physiological pH (7.0), and removal of water molecules beyond a distance of 5 Å from the protein’s natural ligand. Additionally, the protein structure was minimized until the heavy atoms reached a root mean square deviation (RMSD) of 0.30 using the OPLS-3e Force Field (Harder et al., 2016). Conformational energies essential for biological function were restored through the adjustment of torsional parameters.

### 2.2 Active site identification and grid-box generation

Understanding the active site of the protein is essential for conducting molecular docking studies in computer-aided drug design (Ko et al., 2005). Co-crystallized ligand binding sites were chosen to define the protein’s active sites (Alam et al., 2021). To identify the active sites, UCSF ChimeraX was used, and residues involved in binding the co-crystallized ligand were visually inspected to determine the active site region. Using the Glide v11.3 module, we constructed a grid box with dimensions of 72 Å^3^. This grid box was strategically positioned at the center of a smaller secondary box measuring 27 Å^3^ in X, Y, and Z coordinates, allowing for accurate docking calculations (Friesner et al., 2006). Adjustments to the van der Waals radius were made with a selected cutoff value of 0.25, and partial atomic charges were scaled to 1.0. Importantly, no consideration was given to rotating groups during this process.

### 2.3 Generation of the Phase database

A total of 170,269 compound retrievals were conducted using the Asinex Chemical Library available from the ZINC database (www.asinex.com) (Asinex.com, 2022—screening libraries (all libraries)—product types— screening libraries, n. d.) Compounds within the pH range of 6–8 were selected, and their 3D structures were downloaded in SDF format. The Phase module, specifically LigPrep and Epik v4.6, was used to generate a Phase database (Dixon et al., 2006; Greenwood et al., 2010). This process involved extending the protonation and tautomeric states of each molecule, and their 3D structures were used to determine ligand chirality. QikProp properties were then assigned, ensuring adherence to Lipinski’s rule of five to filter potential ligands (Lipinski et al., 2012). Notably, reactive functional groups were excluded during the generation of the Phase database to mitigate false-positive results (Karthikeyan and Vyas, 2014).

### 2.4 High-throughput virtual screening

In our study, we conducted HTVS to efficiently identify potential drug-like components from a large ligand library database (Dhasmana et al., 2019). This approach is particularly effective in searching for compounds that can interact with the protein’s AS (Subramaniam et al., 2008). For ligand-based virtual screening, we used the HTVS protocol within the Glide module. Parameters were set to restrict the maximum number of atoms and rotatable bonds to 500 and 100, respectively. Default settings were applied for van der Waals radius scaling, and post-docking reduction was performed, limiting the number of poses per ligand to a maximum of five.

### 2.5 Molecular docking for binding score analysis

Molecular docking is a computational technique used to predict the binding mode and affinity of small molecules (ligands) to their target proteins. This method aids in identifying potential drug candidates by assessing the strength of interactions between the ligand and the protein (Kandakatla and Ramakrishnan, 2014). In recent years, molecular docking has become an invaluable tool in computational drug discovery within structural biology (Khamouli et al., 2022). In our study, we used the Glide v11 module to investigate the binding mechanism of the target protein with the screened ligands (Friesner et al., 2004; Halgren et al., 2004). The molecular docking procedure was conducted using both standard precision (SP) and extra-precision (XP) modes. These modes enable the evaluation of interactions based on various scoring functions, with the aim of identifying ligands with the highest affinity for the target protein. Visual examination of the protein–ligand complexes and their associated chemical interactions was facilitated using the Maestro Viewer. This analysis provided insights into the specific binding modes and key interactions driving the binding affinity between the ligands and the target protein.

#### 2.5.1 Molecular docking validation

To validate the docking protocol, the co-crystallized inhibitor vandetanib (PDB ID: 2IVU) was redocked into the active site of the pRET tyrosine kinase using Glide software. The protein structure was prepared using the Schrodinger Protein Preparation Wizard, ensuring correct protonation states, hydrogen bonding, and proper orientation of side chains. Initially, vandetanib was prepared using LigPrep to generate its 3D structure and optimize its conformational states. A grid box was generated around the active site of pRET tyrosine kinase, encompassing the co-crystallized ligand binding site. The prepared vandetanib ligand was docked into the grid box using the Glide docking algorithm with SP parameters. The resulting docked pose of vandetanib was compared with its co-crystallized pose in the protein structure. The RMSD between the docked and co-crystallized poses was calculated to assess the accuracy of the docking protocol.

### 2.6 Post-docking MM-GBSA calculation

To investigate the free binding energies of the protein–ligand complexes, we performed an MM-GBSA analysis (Borkotoky et al., 2016). This approach allows for the analysis and visualization of compounds with the lowest binding energy and was implemented using Maestro v12.5.139 and Glide v8.8 (Borkotoky and Banerjee, 2020). The MM-GBSA score was calculated using the Prime MM-GBSA v3.059 package. This method integrates OPLS engineering molecular mechanics (EMM) energies with a VSGB polar solvation model (GSGB) and a nonpolar solvation term (GNP), which includes nonpolar solvent-accessible surface areas (SASAs). The MM-GBSA score, along with docking scores, served as a benchmark for assessing the utility of newly screened drugs. Using Maestro v12.5.13948, we analyzed binding interactions, identified residues involved, and calculated binding free energy (Vijayakumar et al., 2014).

### 2.7 Pharmacokinetics and toxicity prediction

Pharmacokinetic (PK) properties play a critical role in screening drug-like molecules and assessing preclinical safety (Saxena et al., 2017). Factors such as absorption, distribution, metabolism, and excretion (ADME) significantly influence the pharmacological and clinical effectiveness of potential drug candidates. Therefore, we used the SwissADME server (http://www.swiss-ame.ch/) to evaluate the pharmacological properties of the ligand molecules (Daina et al., 2017). This platform assesses the compounds’ compliance with Lipinski’s rule of five, gastrointestinal (GI) permeability, and blood–brain barrier (BBB) penetration, all of which are essential considerations in rational drug design (Lipinski et al., 2001).

Additionally, it is important to assess the potential toxicity of small molecules, as they can pose risks to various organs in the human body, including cytotoxicity, carcinogenicity, hepatotoxicity, immunotoxicity, and mutagenicity. Thus, toxicity prediction is a crucial aspect of drug development. In our study, we used ProTox-II (https://tox-new.charite.de/) to predict the toxicity of the selected compounds (Banerjee et al., 2018).

### 2.8 Molecular dynamics simulation

To comprehensively understand the behavior and stability of protein–ligand complexes in complex biological environments, MD simulations are indispensable (Alam et al., 2021). These simulations provide dynamic insights into the interactions between the ligands and proteins, crucial for assessing binding stability and activity within the active site cavity (Samad et al., 2022). In our study, we conducted MD simulations spanning 150 nanoseconds using the Desmond module from Schrödinger (Release 2020-3) in a Linux environment (Bowers et al., 2006). This allowed us to observe the atomic movements of ligands within protein molecules and capture the complex dynamics of the system. To accurately represent the membrane environment, we employed the TIP3P water model to maintain appropriate water density and electrical permittivity. The system was neutralized with Na^+^ and Clˉ ions to maintain a physiological salt concentration of 0.15 M. Periodic boundary conditions were applied with an orthorhombic box shape (10 × 10 × 10 Å^3) to maintain a constant volume. MD simulations were performed under NPT ensemble conditions at 300 K, 1.01325 bar pressure, and 1.2 ns. Interactions within the solvated protein–ligand system were described using the OPLS-2005 Force Field, with molecules reattached every 15 picoseconds (Harder et al., 2016). These conditions ensured an accurate representation of the biological system and provided information regarding the dynamics of protein–ligand interactions.

## 3 Results

### 3.1 Phase database creation and HTVS

The Phase database creation process involved rapidly screening a compound library to facilitate efficient virtual screening. Using the Phase module of the Asinex Chemical Library, we curated a database comprising 170,269 compounds with pH levels ranging from 6 to 8. From a total of 509,809 conformations generated using the LigPrep function, 98,189 compounds passed through rigorous filtering criteria, including the Lipinski filter and the reactive filter. These filters ensured the selection of compounds meeting essential drug-like properties while minimizing the presence of reactive functional groups. Our results demonstrate the successful establishment of a comprehensive database containing numerous chemical compounds in their optimal conformations. Subsequent docking of molecules from the Phase database into the active site of RET, facilitated by grid-box construction, enabled HTVS. This screening identified 2,550 compounds as potential candidates capable of binding to the target cavity with high affinity.

### 3.2 Molecular docking for binding score analysis

**FIGURE 2:**
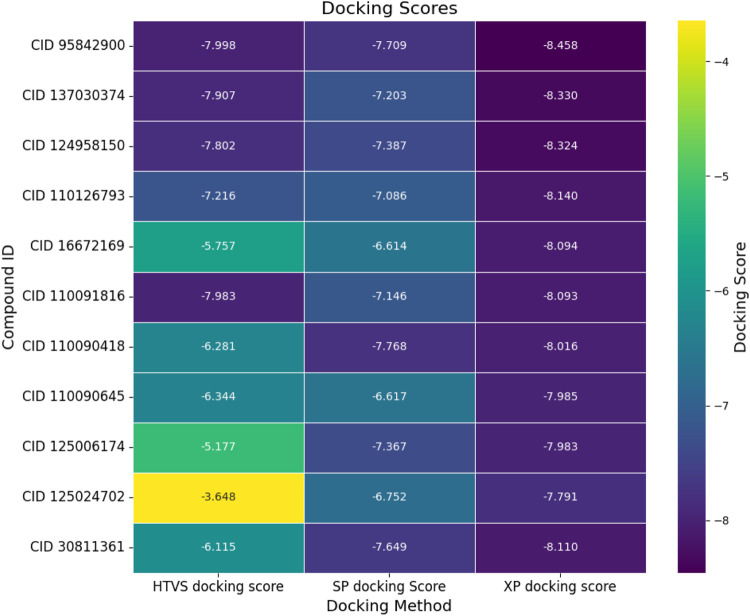
Molecular docking scores (kcal/mol) of the top ten hit compounds and native ligand evaluated through different docking approaches. Here, the first column represents the high-throughput virtual screening (HTVS), the second column represents the standard precision (SP), and the third column represents the extra precision (XP) docking scores for respective compounds. The yellow to blue color denotes the elevation of the negative binding score.

Molecular docking is a powerful computational method used to predict the binding affinity between a ligand and a protein within a complex, providing valuable insights for optimizing potential drug candidates. In this study, we used various molecular docking approaches, including HTVS, SP docking, and XP docking, to evaluate the binding interactions of small-molecule compounds with the target protein. The analysis identified the top 10 intramolecular chemical structures based on their docking scores shown in Figure 2 and listed in Supplementary Figure S1. These compounds, along with the native ligand, were further scrutinized for their binding affinities. The XP docking approach revealed a binding score ranging from −7.791 kcal/mol to −8.458 kcal/mol across the selected compounds.

From the comprehensive docking analysis, four compounds exhibited superior binding affinities compared to the control compound (vandetanib), as shown in Figure 2. Specifically, these lead compounds demonstrated binding scores of −8.458 kcal/mol, −8.33 kcal/mol, −8.324 kcal/mol, and −8.14 kcal/mol, respectively. Vandetanib (CID 30811361), serving as the native ligand, exhibited a binding score of −8.11 kcal/mol.

#### 3.2.1 Docking validation

**FIGURE 3:**
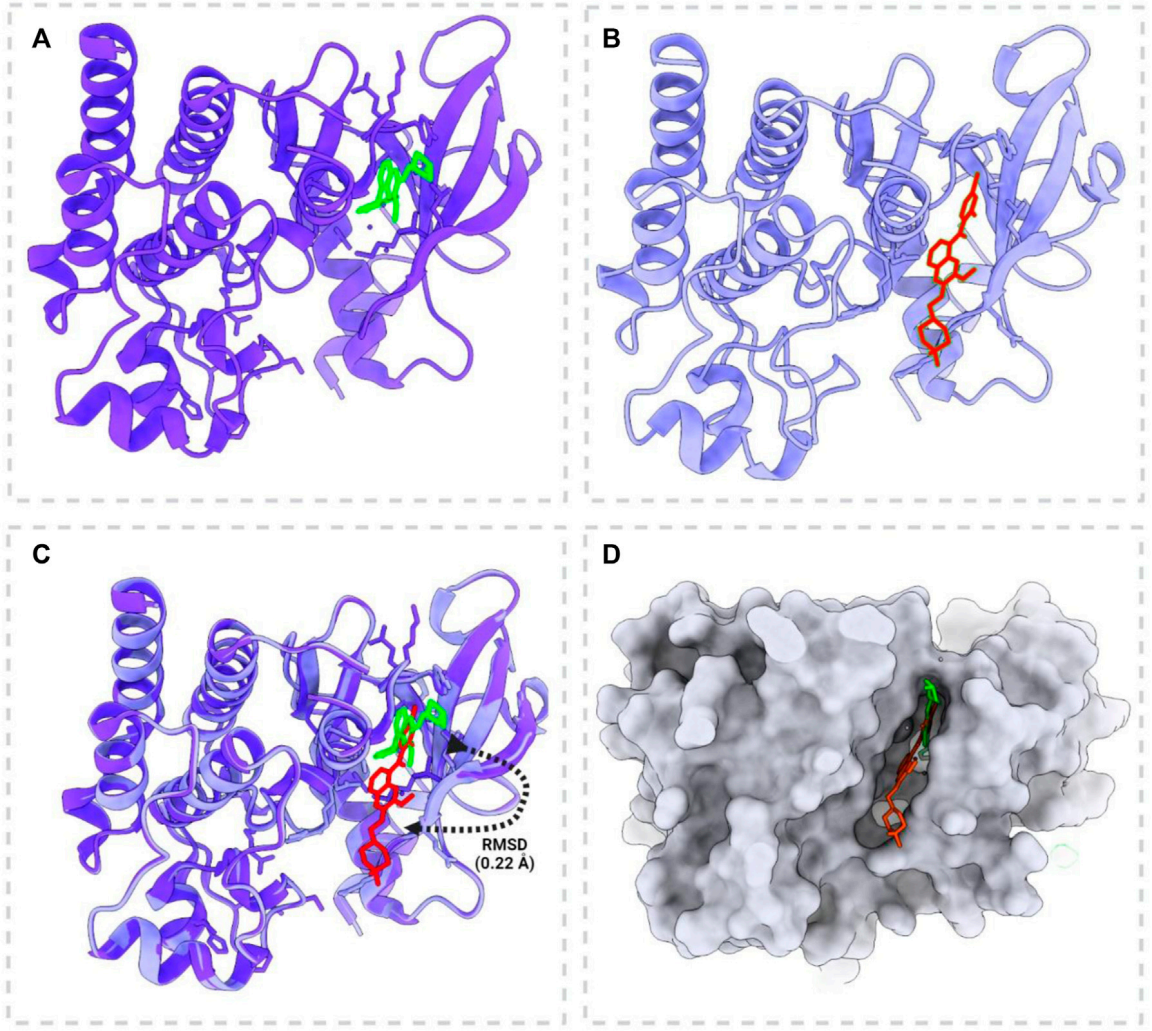
Docking validation of vandetanib with phosphorylated RET tyrosine kinase (PDB: 2IVU). **(A)** Native complex form of the crystal structure of phosphorylated RET tyrosine kinase bound to vandetanib. **(B)** Redocking position of vandetanib with phosphorylated RET tyrosine kinase, demonstrating the docking process. **(C)** Superimposition of the crystal structure and the docked model, showing the root mean square deviation (RMSD) to illustrate the accuracy of the docking process. **(D)** Active site pocket and surface view highlighting vandetanib in the active pocket before docking (red) and after docking (green). This panel demonstrates the changes in vandetanib’s position and conformation within the binding site after the docking procedure.

**FIGURE 4:**
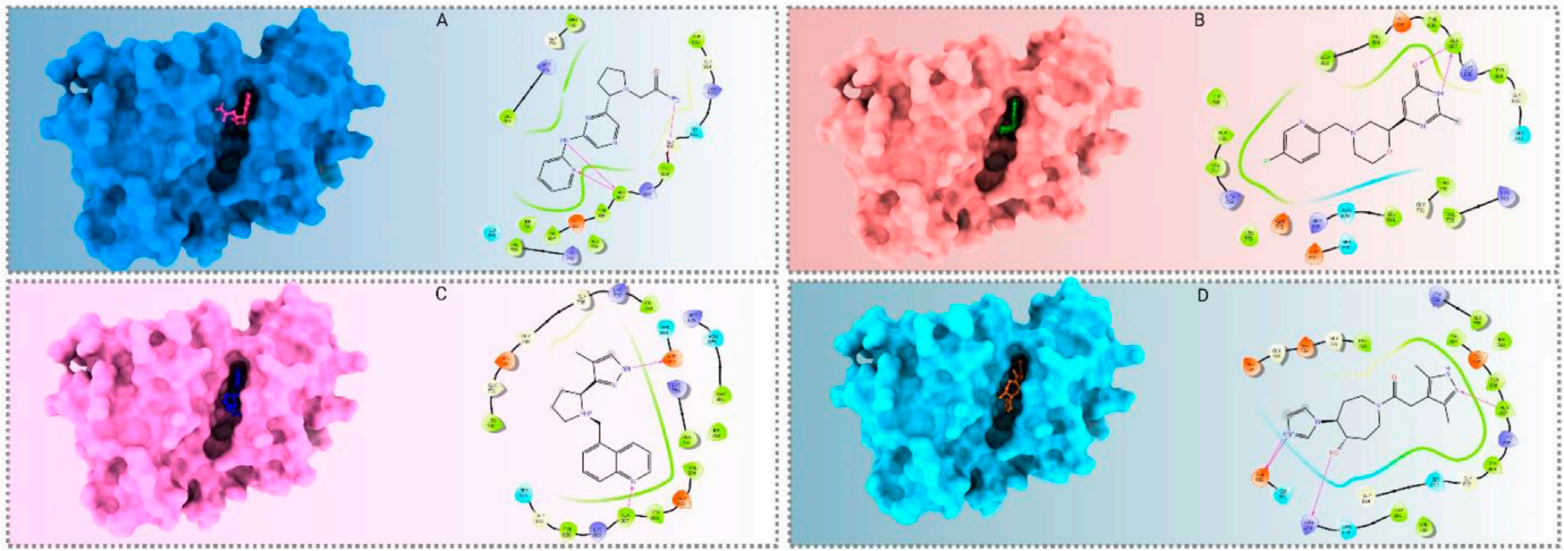
Molecular docking interactions between the pRET tyrosine kinase and the four selected compounds, presented in both 3D and 2D formats. **(A–D)** Interactions of CID 95842900, CID 137030374, CID 124958150, and CID 110126793, respectively.

The redocking of vandetanib into the active site of pRET tyrosine kinase yielded a docked pose that closely resembled its co-crystallized pose (Figure 3). The RMSD between the docked and co-crystallized poses was 0.22 Å, which is within an acceptable range, indicating the reliability of the docking protocol (Figures 4A–D). This validation procedure confirms the suitability of Glide software for docking new compounds into the active site of pRET tyrosine kinase for further virtual screening studies.

### 3.3 Protein–ligand binding interaction

**FIGURE 5:**
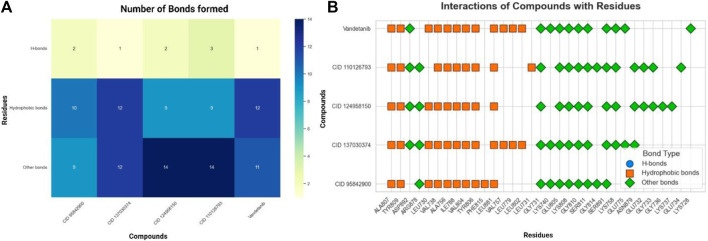
Residual interactions and proximity analysis of the selected four compounds. **(A)**. Various types and numbers of residual interactions observed between the four selected compounds and vandetanib, the control compound. Each bar indicates the count of specific interaction types and shows how the selected compounds differ in their binding characteristics compared to vandetanib. **(B)**. The residues unique to proteins and the selected four compounds are presented alongside the types of contacts established. The closeness of these compounds to proteins identifies the specific residues involved in their interactions.

Binding studies were conducted using Maestro (v12.5) to visualize the interaction bonds with residues, as shown in Figure 4. The interactions between the ligands and the target protein were analyzed, and the results are presented in Figure 5 and Supplementary Table S1. These interactions revealed a diverse array of non-bonded interactions, including hydrogen bonds, hydrophilic and hydrophobic interactions, electrostatic links, and polar bonds.

The presence of hydrogen bonds in the interactions was found to contribute significantly to the stability of the protein–ligand complex. Specifically, CID 95842900, CID 124958150, and CID 110126793 exhibited two hydrogen bonds, while CID 137030374 and vandetanib demonstrated only one hydrogen bond (Figure 5A). Interestingly, compared to the control drugs, the three ligands showed a higher number of hydrogen bonds. Remarkably, all selected compounds interacted with a common residue, ALA807, via hydrogen bonding. Additionally, several other residues, including LEU730, GLY731, VAL738, ALA756, ILE788, VAL804, GLU805, TYR806, LYS808, TYR809, GLY810, SER811, LEU881, and SER891, were identified as common interacting residues (Figure 5B). These residues engage in various types of bonds, such as hydrophobic, glycine, charged, and polar bonds.

### 3.4 Post-docking MM-GBSA analysis

**FIGURE 6:**
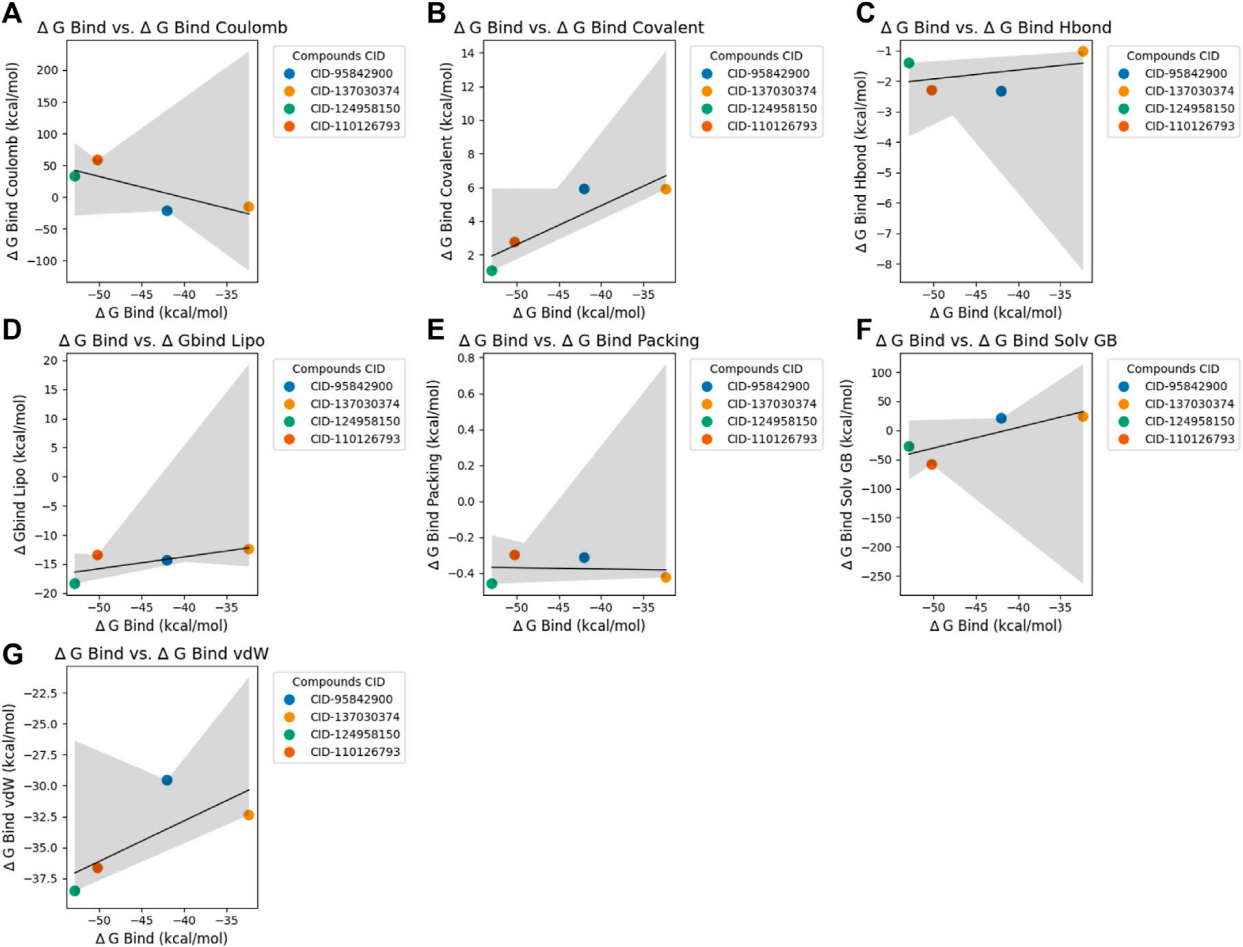
Scatter plots depicting the relationship between ∆ G Bind (binding energy) and various binding energies for selected four compounds. Each panel corresponds to a specific type of binding energy and shows its correlation with ∆ G Bind for the chosen compounds, which are distinguished by different colors. The black line in each panel represents the linear regression fit for the data points. Plot **(A)**: ∆ G Bind vs. ∆ G Bind Coulomb. Plot **(B)**: ∆ G Bind vs. ∆ G Bind Covalent. Plot **(C)**: ∆ G Bind vs. ∆ G Bind H-bond. Plot **(D)**: ∆ G Bind vs. ∆ Gbind Lipo. Plot **(E)**: ∆ G Bind vs. ∆ G Bind Packing. Plot **(F)**: ∆ G Bind vs. ∆ G Bind Solv GB. Panel **(G)**: ∆ G Bind vs. ∆ G Bind vdW. This visualization aids in understanding the relationship between ∆ G Bind and various binding energies for the compounds under study.

The MM-GBSA calculations were conducted to evaluate the binding free energy of selected four compounds, namely, CID 95842900, CID 137030374, CID 124958150, and CID 110126793 (Figure 6), and the top 10 hit binding scores are exhibited in Supplementary Table S2 and Supplementary Figure S2. The compound CID 95842900 binding free energy is estimated to be approximately −41.98 kcal/mol. This value comprises various energy components, including Coulombic interaction energy (∆G_bind Coulomb) of −21.93 kcal/mol, covalent interaction energy (∆G_bind Covalent) of 5.90 kcal/mol, hydrogen bonding energy (∆G_bind Hbond) of −2.34 kcal/mol, lipophilic interaction energy (∆G_bind Lipo) of −14.36 kcal/mol, packing interaction energy (∆G_bind Packing) of −0.31 kcal/mol, solvation energy (∆G_bind Solv GB) of 20.62 kcal/mol, and van der Waals interaction energy (∆G_bind vdW) of −29.56 kcal/mol (Figures 6A–G). The compound CID 137030374 shows a binding free energy of −32.33 kcal/mol. It is comprised of Coulombic interaction energy of −15.63 kcal/mol, covalent interaction energy of 5.88 kcal/mol, hydrogen bonding energy of −1.03 kcal/mol, lipophilic interaction energy of −12.44 kcal/mol, packing interaction energy of −0.42 kcal/mol, solvation energy of 23.69 kcal/mol, and van der Waals interaction energy of −32.38 kcal/mol (Figures 6A–G). The binding free energy for CID 124958150 is approximately −52.90 kcal/mol. This value includes Coulombic interaction energy of 32.60 kcal/mol, covalent interaction energy of 1.03 kcal/mol, hydrogen bonding energy of −1.41 kcal/mol, lipophilic interaction energy of −18.39 kcal/mol, packing interaction energy of −0.46 kcal/mol, solvation energy of −27.77 kcal/mol, and van der Waals interaction energy of −38.51 kcal/mol (Figures 6A–G). The calculated binding free energy for CID 110126793 is −50.20 kcal/mol. It consists of Coulombic interaction energy of 58.40 kcal/mol, covalent interaction energy of 2.75 kcal/mol, hydrogen bonding energy of −2.30 kcal/mol, lipophilic interaction energy of −13.45 kcal/mol, packing interaction energy of −0.30 kcal/mol, solvation energy of −58.66 kcal/mol, and van der Waals interaction energy of −36.64 kcal/mol.

### 3.5 Pharmacokinetics and toxicity properties’ analysis

Drug discovery and development necessitate a thorough understanding of pharmacokinetic features, including absorption, distribution, metabolism, excretion, and toxicity. Predicting pharmacokinetic properties is crucial in computational drug discovery as it aids in understanding preclinical failures and drug distribution within the human body (Lipinski et al., 2012). Toxicology prediction is an essential aspect of drug development regulation, as it informs us about the potential harm that chemicals may pose to humans, animals, and the environment. In this study, the four compounds with the highest binding affinity, namely, CID 95842900, CID 137030374, CID 124958150, and CID 110126793, along with the control drug vandetanib, were screened for ADME and toxicity.

**FIGURE 7:**
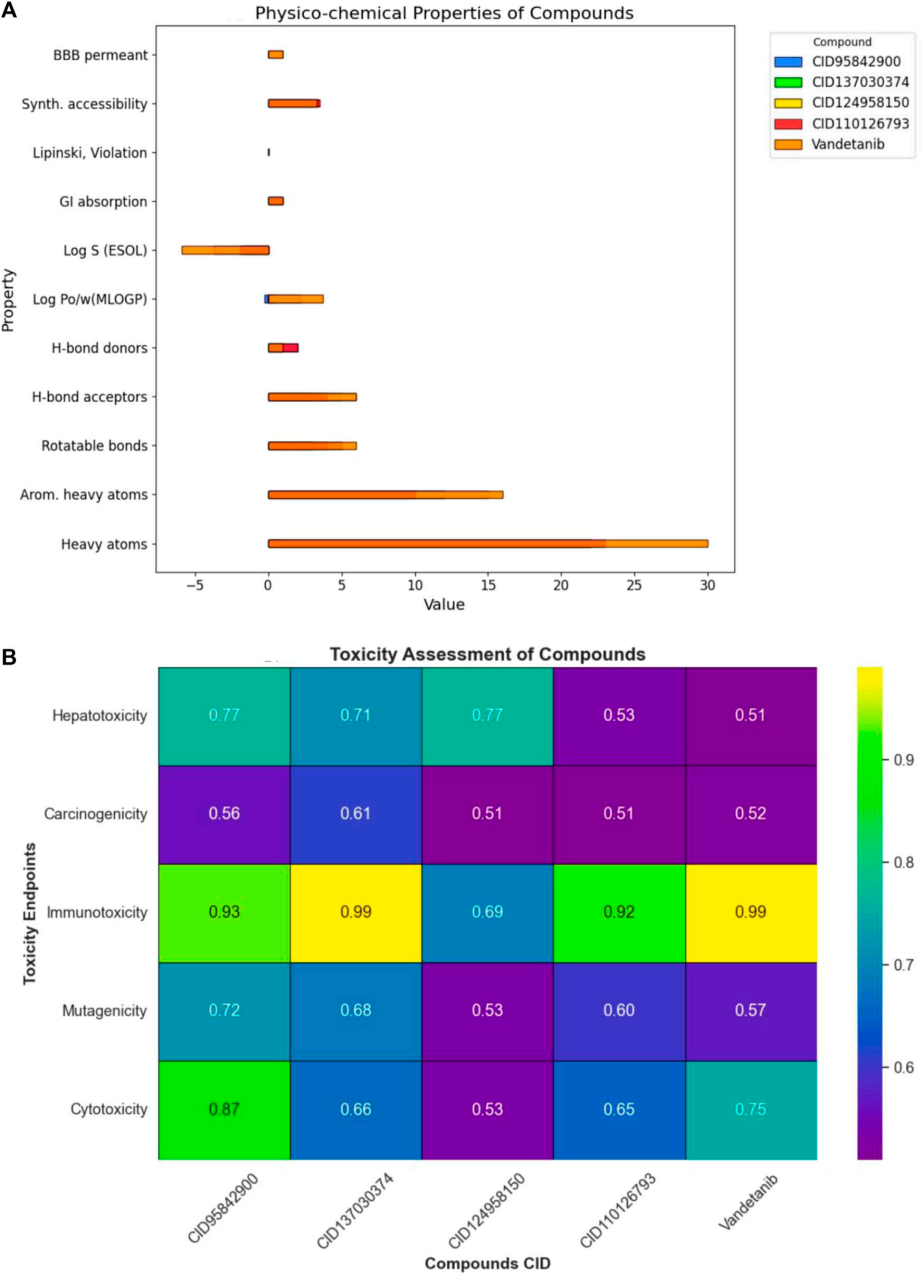
Overview of pharmacokinetic properties for selected compounds. Herein, **(A)** provides a comprehensive assessment of various pharmacokinetic properties of the four selected compounds. It covers a wide range of parameters including physicochemical properties, lipophilicity, water solubility, pharmacokinetics, drug-likeness, medicinal chemistry and blood-brain barrier (BBB) permeability. Where, **(B)** represents the parameters such as, hepatotoxicity, carcinogenicity, immunogenicity, mutagenicity, and cytotoxicity.

The ADMET predictions for these compounds are presented in Figures 7A, B and listed in Supplementary Tables S3, S4. The selected compounds were found to comply with Lipinski’s rule, indicating favorable pharmacokinetic properties. They exhibited high GI tract permeability, suggesting enhanced bioavailability at the target site (Figure 7A). Moreover, these compounds showed no signs of hepatotoxicity, carcinogenicity, immunogenicity, mutagenicity, or cytotoxicity. Consequently, the selected compounds were deemed non-toxic to humans and categorized into toxicity classes III, IV, III, IV, and IV, respectively (Figure 7B).

### 3.6 Molecular dynamics simulations

The MD simulation can predict the thermodynamic stability that investigates the authenticity of the post-molecular docking study. In our study, we used MD simulations with an orientation time of 150 ns to analyze the trajectories of the top four compounds, with a native inhibitor (vandetanib) as the control compound and one control structure (apo) to determine the actual motion of atoms and macromolecules. Analysis of the trajectory and pose in this MD simulation was performed using several different metrics, including RMSD of protein and ligand, root mean square fluctuations (RMSF), radius of gyration (rGyr), SASA, molecular surface area (MolSA), polar surface area (PSA), hydrogen bond analysis, ligand torsions, protein–ligand contact, and ligand–protein contact.

#### 3.6.1 RMSD analysis of proteins

**FIGURE 8:**
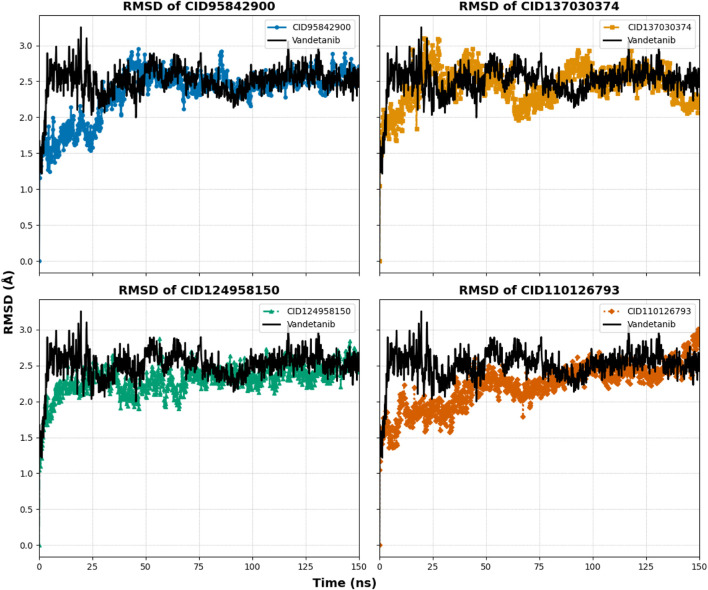
Graphs representing the MD simulation for the selected protein–ligand complexes, focusing on protein Cα RMSD over a 150 ns simulation period. The compounds CID 95842900, CID 137030374, CID 124958150, and CID 110126793 are depicted in blue, yellow, green, and orange, respectively, in comparison to the control compound vandetanib (black).

The RMSD analysis was conducted to assess the equilibrium state of the MD simulation. RMSD calculates the average shift in atoms between frames, indicating structural changes in the protein–ligand complex over time (Opo et al., 2021). In this study, the average RMSD variation for the protein–ligand complexes remained within an acceptable range of 1–3 Å (Alam et al., 2021). Deviations beyond this range suggest substantial conformational shifts in the protein structure, indicating whether the chosen ligand–protein complexes are appropriately compared with the apo protein control. To evaluate the structural consistency of the selected complexes (CID 95842900, CID 137030374, CID 124958150, and CID 110126793), RMSD values were compared with both the apo-form and the control–drug complex (vandetanib) over a 150 ns simulation period, as shown in Table 1 and Figure 8, and Supplementary Figure S3. These complexes exhibited average RMSD values of 2.352 Å, 2.442 Å, 2.301 Å, and 2.217 Å, respectively, compared to the apo structure, and 2.303 Å and 2.49 Å, respectively, compared to the control compound. Notably, all selected compound complexes showed lower fluctuations than the control complexes and overlapped with the apo protein.

**TABLE 1:** Four lead compounds, along with the control drug (vandetanib), generated different parameters including the highest, lowest, and average values from the 150 ns molecular dynamics simulation.

Parameter	Value	Apo	CID 95842900	CID 137030374	CID 124958150	CID 110126793	Vandetanib
Protein Cα RMSD	H. RMSD (Å)	3.27	2.949	3.101	2.874	3.005	3.253
	L. RMSD (Å)	1.24	1.15	1.04	1.04	1.05	1.21
	A. RMSD (Å)	2.3	2.35	2.44	2.3	2.21	2.49
Ligand Cα RMSD	H. RMSD (Å)	N/A	4.443	7.484	9.744	2.101	2.35
	L. RMSD (Å)	N/A	0.543	1.032	0.426	0.485	0.581
	A. RMSD (Å)	N/A	2.39	4.52	2.96	1.07	1.41
Protein Cα RMSF	H. RMSD (Å)	11.9	6.377	7.043	9.35	8.083	8.743
	L. RMSD (Å)	0.38	0.41	0.423	0.369	0.353	0.379
	A. RMSD (Å)	1.08	1.05	1.15	1	0.99	1.02
Radius of gyration	H. RMSD (Å)	N/A	3.951	4.499	3.786	4.22	6.125
	L. RMSD (Å)	N/A	3.497	3.374	3.018	3.617	5.712
	A. RMSD (Å)	N/A	3.69	4.01	3.56	3.94	5.95
Solvent-accessible surface area	H. RMSD (Å^2^)	N/A	182.57	238.636	314.04	117.456	232.198
	L. RMSD (Å^2^)	N/A	77.59	55.327	55.942	44.368	117.691
	A. RMSD (Å^2^)	N/A	141.85	132.65	143.33	73.1	181
Molecular surface area	H. RMSD (Å^2^)	N/A	296.937	293.454	297.316	315.317	410.737
	L. RMSD (Å^2^)	N/A	280.284	271.442	263.236	295.858	393.956
	A. RMSD (Å^2^)	N/A	291.1	285.56	289.7	308.88	405.13
Polar surface area	H. RMSD (Å^2^)	N/A	173.101	116.514	74.952	151.03	151.04
	L. RMSD (Å^2^)	N/A	143.236	81.359	55.799	124.92	124.93
	A. RMSD (Å^2^)	N/A	162.89	102.72	65.65	136.71	136.72

H, highest; L, lowest; A, average.

The compound CID 95842900 displayed a maximum RMSD of 2.949 Å and a minimum RMSD of 1.155 Å, as shown in Figure 8. Similarly, the CID 137030374 complex exhibited a maximum RMSD of 3.101 Å and a minimum RMSD of 1.047 Å. The CID 124958150 complex exhibited a maximum RMSD of 2.874 Å and a minimum RMSD of 1.044 Å, while Figure 8 depicts the CID 110126793 complex with corresponding maximum and minimum RMSD values of 3.279 Å and 1.051 Å. Compared to the apo structure, the highest and lowest RMSD values were 3.005 Å and 1.241 Å, respectively (Table 1). When compared to the control ligand vandetanib, the highest and lowest RMSD values were 3.253 Å and 1.216 Å, respectively, as shown in Figure 8. All complexes exhibited significant similarities between their maximum and minimum RMSD values, comparable to the vandetanib structure, indicating less deviation from the control ligand. Furthermore, the selected complexes demonstrated stability after 30 ns of simulation, with deviations similar to the apo structure, while the control complexes exhibited larger deviations until the 90 ns timeframe.

#### 3.6.2 Ligand fit protein RMSD

**FIGURE 9:**
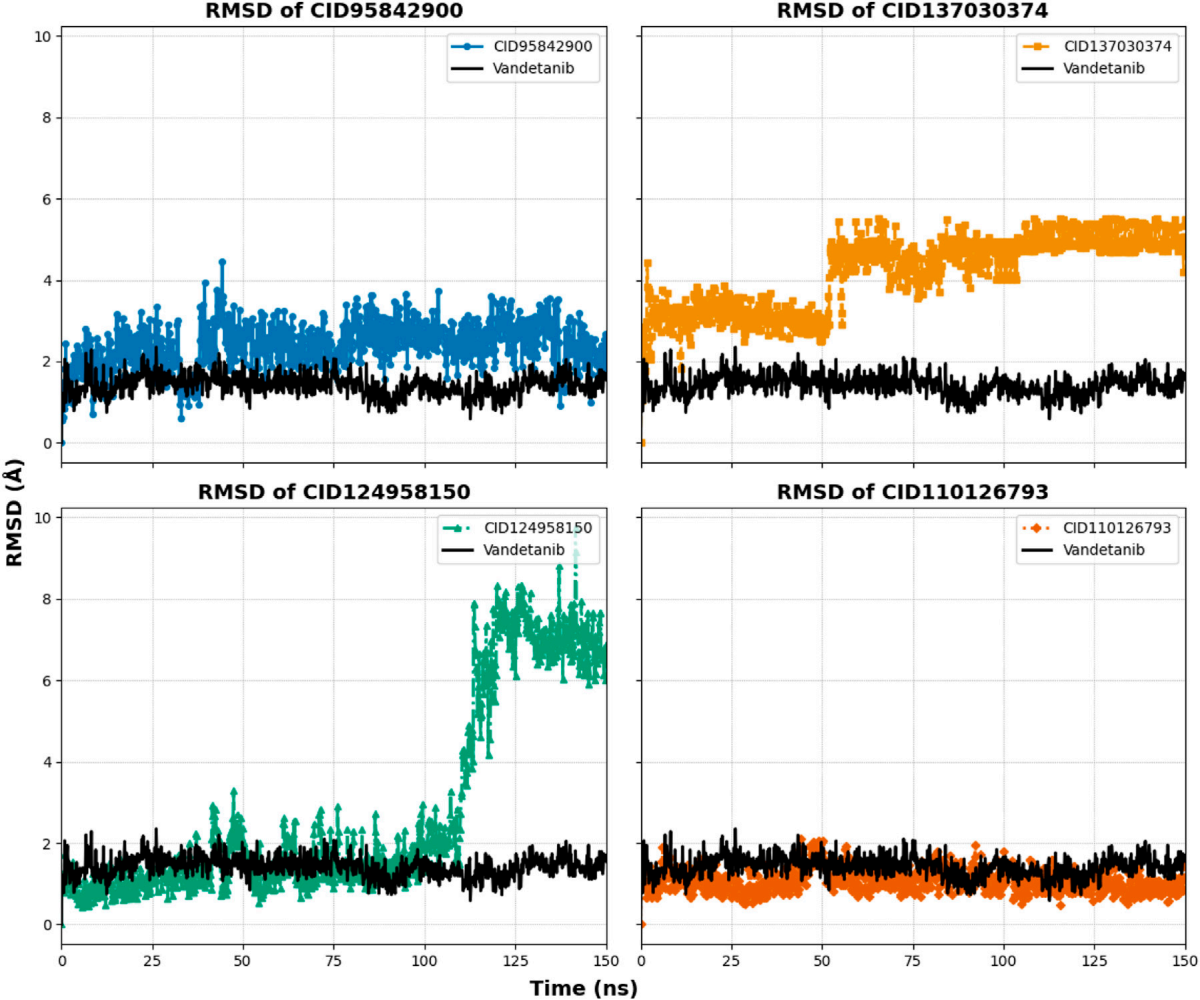
RMSD values extracted for protein–ligand complex alpha carbon (Cα) atoms of the selected four compounds (ligands) during a 150 ns simulation period. The compounds CID 95842900, CID 137030374, CID 124958150, and CID 110126793 are represented in blue, yellow, green, and orange, respectively, while the control compound vandetanib is depicted in black for comparison.

Ligand fit protein Cα RMSD of 150 ns simulation was analyzed to indicate the ligand being stable when bound to the protein binding site. The four selected complexes (CID 95842900, CID 137030374, CID 124958150, and CID 110126793) and the control compound complex (vandetanib) (black) had calculated average values of 2.390 Å, 4.524 Å, 2.966 Å, 1.073 Å, and 1.410 Å, respectively, as illustrated in Table 1 and Figure 9, and Supplementary Figure S4. From this evaluation, CID 137030374 shows significant diffusion from the control drug complexes, CID 95842900 and CID 110126793 exhibit less deviation, and CID 124958150 overlaps with the control drug complexes from the start to the end of the 150 ns simulation.

#### 3.6.3 RMSF analysis

**FIGURE 10:**
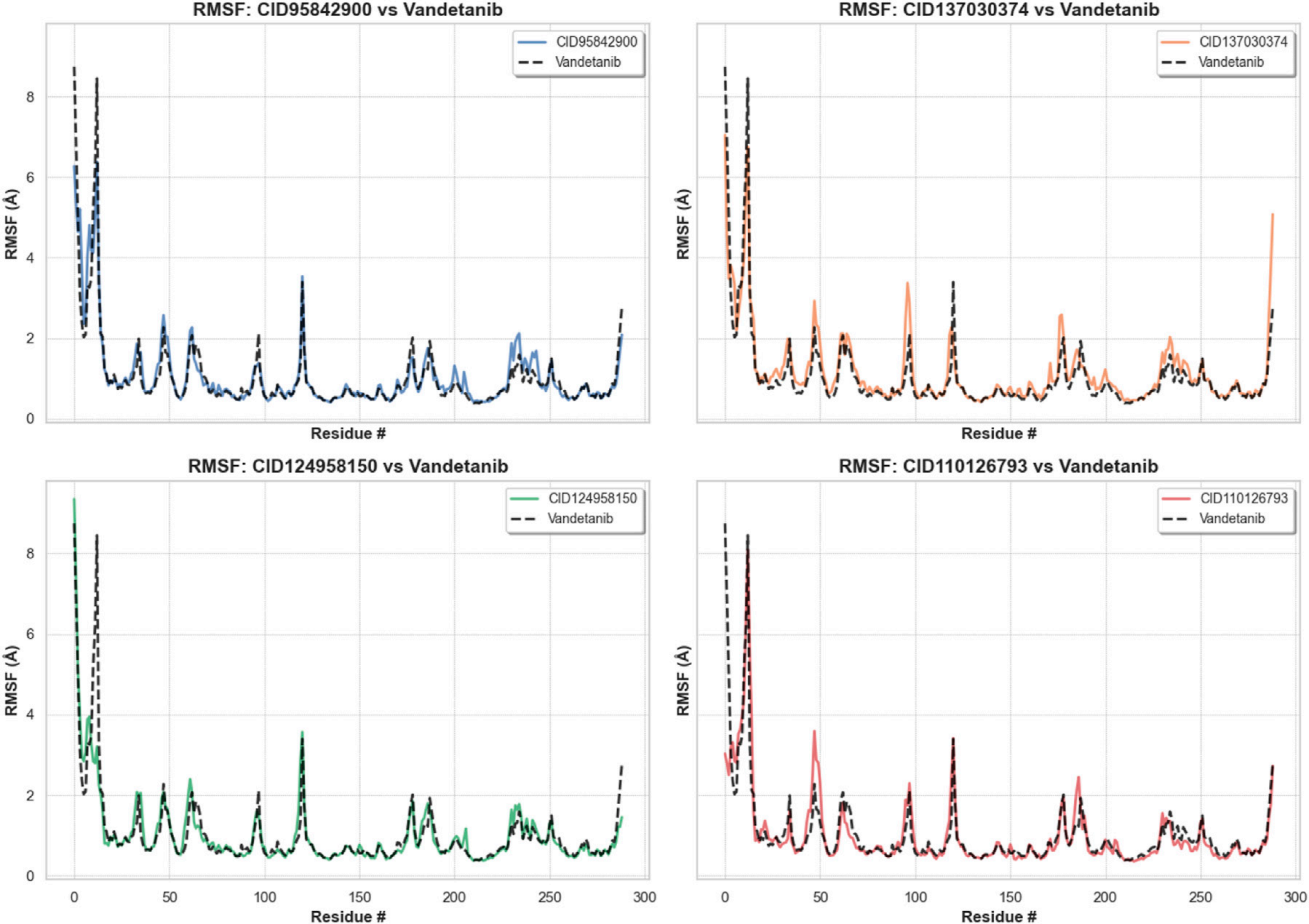
RMSF values of pRET tyrosine kinase were reclaimed from protein Cα atoms of the protein–ligand docked complexes. The compounds CID 95842900, CID 137030374, CID 124958150, and CID 110126793 are represented in blue, yellow, green, and orange, respectively, while the control compound vandetanib is depicted in black for comparison.

The RMSF allows us to determine macromolecule heterogeneity and steady state and provides insight into the local conformational changes in amino acid residues (Bharadwaj et al., 2021). Figure 10 shows that the largest changes happened in the residual positions of LEU712, GLU734, LEU746, GLU762, SER795, SER819, GLU901, and GLN910. All of the compounds’ amino acid residues showed the least amount of change compared to the apo protein, except LEU712 from the vandetanib (control), which showed the biggest change of 8.446 Å. All of the other compounds showed the least amount of change. The average fluctuation was 1.056 Å, 1.158 Å, 1.009 Å, 0.998 Å, 1.008 Å, and 1.002 Å for CID 95842900, CID 137030374, CID 124958150, and CID 110126793, compared with the control compound, vandetanib (native drugs), respectively, as shown in Table 1 and Supplementary Figure S5. When compared with apo and control compounds, the average fluctuation of the selected complexes was the least, indicating that the protein complex was the most flexible without altering its macromolecular structure.

#### 3.6.4 Radius of gyration analysis

The rGyr is the square of the radial distance between the center of mass of the targeted protein–ligand complexes, and it is used to quantify the stiffness and mobility of a protein at its terminal (Molla et al., 2023). It is the usual shift in protein–ligand compactness due to macromolecule structural activity. Therefore, in this 150 ns simulation, rGyr is explored for CID 95842900 (red), CID 137030374 (orange), CID 124958150 (gray), and CID 110126793 (yellow), compared with control structures of the vandetanib (control) (green), as presented in Table 1, and Supplementary Figure S6 shows the average rGyr values of 3.694 Å, 4.015 Å, 3.566 Å, 3.940 Å, and 5.952 Å, respectively. The rGyr outcome shows that all the selected complexes exhibit a lower radial distance than control drugs. In Supplementary Figure S6A, the rigidity measurements of CID 95842900 (red) and CID 110126793 (yellow) are approximately less than 1 Å, where CID 137030374 (orange) shows a large peak for 5 ns time after 100 ns simulation and CID 110126793 (yellow) measured a smaller peak at 80–85 ns. Except for this time mentioned, all the complexes indicate that the receptor’s active site does not undergo any significant conformational changes after binding with the ligands.

#### 3.6.5 Surface area analysis

The measurement of the surface area is essential in comprehending the dynamic nature of ligand–protein binding. It additionally provides information regarding the optimization of a site, the identification of the binding site, and the accessibility of ligands in macromolecular environments (Imon et al., 2023). In this study, SASA, MolSA, and PSA of the surface area were carried out over the course of a 150 ns simulation of proteins in complex with the four lead compounds, namely, CID 95842900 (red), CID 137030374 (orange), CID 124958150 (gray), and CID 110126793 (yellow). These were compared with the control ligand vandetanib (green), and the results were calculated and are presented in Table 1 and Supplementary Figure S6. Supplementary Figure S6B shows that the minimal and maximal SASA values were calculated as (77.59-182.57) Å^2^ for CID 95842900, (55.327-238.636) Å^2^ for CID 137030374, (55.942-314.04) Å^2^ for CID 124958150 (gray), and (44.368-117.456) Å^2^ for CID 110126793, and in comparison, the control drug complex vandetanib exhibited SASA values of (117.691-232.198) Å^2^, indicating that the amino acids of the targeted protein have lower exposure from the active site when complexed with the lead compounds (Table 1). Supplementary Figure S6C shows that the average MolSA values of the lead compounds are 291.106 Å^2^, 285.565 Å^2^, 289.704 Å^2^, and 308.889 Å^2^, respectively, while the control drug has 405.139 Å^2^. The four lead compounds exhibit a lower molecular surface area than control drugs, indicating that the protein–ligand complexes facilitate an in-depth understanding of their flexible and adaptable nature over the 150 ns simulation time. Finally, in Supplementary Figure S6D, the PSA analysis showed the average values of 162.899 Å^2^, 102.721 Å^2^, 65.65 Å^2^, 136.713 Å^2^, and 48.974 Å^2^ for CID 95842900 (red), CID 137030374 (orange), CID 124958150 (gray), CID 110126793 (yellow), and control ligand vandetanib, respectively (Table 1). The control drug has lower PSA values, whereas the PSA values of lead compounds are also within an acceptable range, except for CID 95842900, which has a larger polar surface area than other compounds. The PSA analysis of the protein–ligand complexes confirmed the stability of these complexes, suggesting strong binding between the protein and the drug molecules.

#### 3.6.6 Hydrogen bond analysis

**FIGURE 11:**
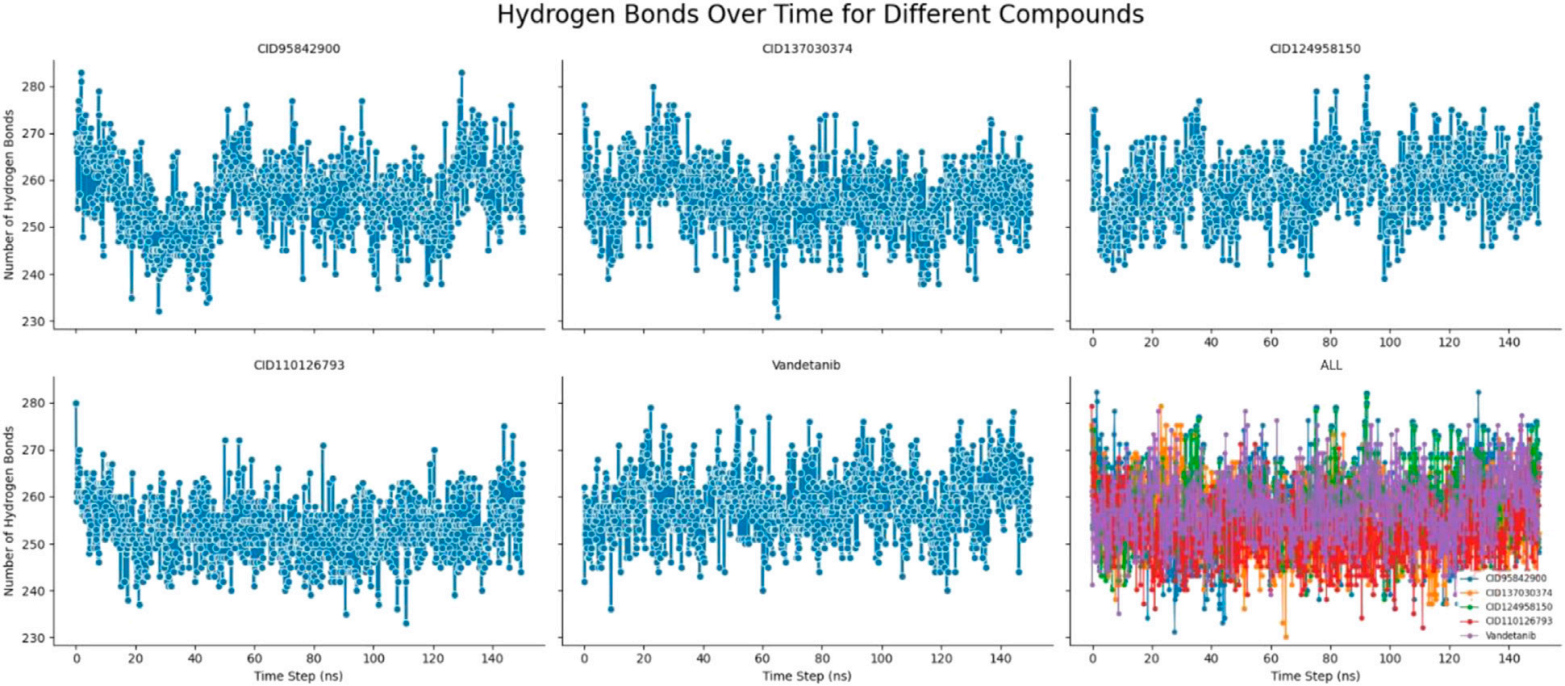
Number of hydrogen bonds formed of the selected four compounds in a complex with the desired pRET tyrosine kinase and control drug complex during the 150 ns molecular dynamics simulation. The last plot represent the combined hydrogen bond number of selected four compounds CID 95842900, CID 137030374, CID 124958150, CID 110126793, and control compounds of vandetanib, respectively.

Hydrogen bonds can help characterize a drug-binding site that plays an acute role in drug and desired protein interaction and influences drug specificity, metabolism, and adsorption (Bharadwaj et al., 2021; Opo et al., 2021). Therefore, the number of hydrogen bonds of the selected four complexes, namely, CID 95842900, CID 137030374, CID 124958150, and CID 110126793, compared with the control ligand vandetanib, was computed for systems by considering conformations every 150 ps, as signified in Figure 11. From the beginning of the 150 ns simulation to the end, the number of hydrogen bonds formed was calculated. Multiple hydrogen bonds, from approximately 230 to 290, formed in each molecule simultaneously. As a result, the ligand–receptor interaction will be considerably strengthened and stabilized by the presence of all the molecules.

#### 3.6.7 Protein–ligand contact analysis

**FIGURE 12:**
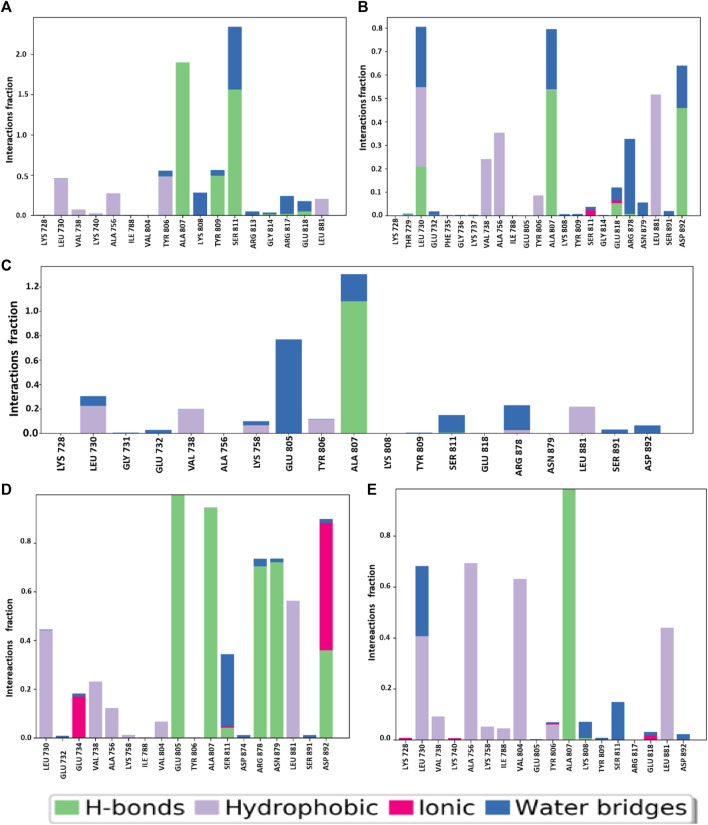
During the 150 ns MD simulation, the various forms of bonding that took place along the protein–ligand interface are illustrated. The four selected compounds **(A)** CID 95842900, **(B)** CID 124958150, **(C)** CID 137030374, **(D)** CID 110126793, and control compounds **(E)** vandetanib are presented.

Using the simulation interaction diagram (SID), we have analyzed the intramolecular interactions between the target protein complex and the selected small biological molecules (CID 95842900, CID 137030374, CID 124958150, and CID 110126793) and the control drug (vandetanib) over the course of the 150-ns MD simulation represented in Figure 12. Many diverse binding interactions are engaged when a protein complex interacts with its ligand. Six different amino acids, LEU730, VAL738, ALA756, TYR806, ALA807, and SER811, have been identified as participating in shared binding interactions between the control and four selected molecules in the protein–ligand contacts. LYS728, LYS808, and TYR809 positions were common in the three ligand and control drugs except for CID 110126793. A stable interaction between the drug and its target protein requires the use of a binding site that is highly conserved. Identical residues were discovered to participate in many binding interactions across all complexes, with high interaction fraction values at common binding locations indicating the presence of hydrogen bonds and other types of bonding. The ligand–protein interaction analysis determined how the ligand atoms strongly bind with protein residues. For all selected ligands and the control drug, the common binding residue was ALA807, indicating that this binding stabilizes throughout the entire 150 ns simulation.

## 4 Discussion

The prevalence of cancer has increased over the past generation, making it the leading cause of death around the world (Thun et al., 2010). Therefore, malignant growth poses a significant threat to human health (Huang et al., 2021). Oncogenesis is frequently associated with the activation of RET, which plays an important role in regulating various oncogenic signaling pathways. The RET gene encodes a protein that is essential for the normal functioning of cells. However, mutations or fusions in RET can result in abnormal proteins, which can fuel uncontrolled cell growth, prevent programmed cell death, and stimulate the growth of new blood vessels feeding the tumor. These alterations contribute to the progression of diverse cancers where RET function is disrupted. In this study, we aim to develop a potential drug candidate from the Asinex ZINC database targeting pRET tyrosine kinase. To explore novel lead compounds, we use high-throughput virtual screening, molecular docking, post-docking MM-GBSA, ADME/T, and MD simulation procedures. HTVS is a method to screen a large number of compounds that are difficult to screen experimentally (Tripathi and Bandyopadhyay, 2022). HTVS revealed 98,189 compounds based on Lipinski filtration and reactive group filtration. Lipinski filtration identifies drug-like compounds, while reactive group filtration focuses on molecules that are particularly reactive or prone to forming bonds with other molecules (Bruns and Watson, 2012). Molecular docking is a key technique that assesses binding affinity and intermolecular interactions at the atomic level between a target protein and bioactive compounds (Daoui et al., 2022). Through this study, we screened 10 biologically active compounds that have promising negative binding energy, indicating a significant interaction with the targeted protein. Among these 10 compounds, four showed an upper binding affinity compared to vandetanib (a native inhibitor) and shared multiple common interactive amino acid residues, confirming possible binding in the active site of the protein, as presented in Supplementary Table S1. The redocking/docking validation protocols accessing the accuracy of molecular docking methods predict the binding pose for new small molecules in the drug discovery process (Parate et al., 2022a). We performed docking validation techniques that provided an acceptable range of RMSD (0.220 Å) and ensured that the hits bind to the protein active site. The MM-GBSA method is employed to compute the binding free energy between a protein and a ligand. In the MM-GBSA study, the lowest ∆G Bind score (the most negative score) represents the best ∆G Bind score (Zhang et al., 2017). In the MM-GBSA analysis, four compounds were found to have higher net negative binding free energy values than the control (−13.537833144 kcal/mol). As MM-GBSA analysis validated the docking score, the obtained outcome suggests that the selected four compounds remain stable in protein–ligand complexes. As a result of the ADME/T analysis, we determined that all four ligands have promising pharmacokinetic properties with no toxicity profile, which suggests the potentiality of being a lead compound (Chtita et al., 2022).

Molecular dynamics simulations are used to determine the stability of a protein when it is complexed with its ligand. Furthermore, it has the ability to determine the stability and rigidity of proteins and their ligands in a specific artificial human body-like environment (Pavan et al., 2022). The MD simulations conducted in this study provided valuable insights into the conformational stability and dynamics of the protein–ligand complexes over a 150 ns timescale. Based on MD simulations, RMSD values are used to calculate the stability of the protein–ligand complex (Coutsias et al., 2004). Cα atoms are crucial parameters of MD simulations. Using this parameter, we can calculate the deviation of the backbone of a single frame in a dynamic environment (Stark et al., 2003). The RMSD analysis confirms the stability of the selected four compounds in an artificial human environment. RMSF is a useful tool for evaluating structural movement and flexibility. Studying protein–ligand interactions relies heavily on monitoring the behavior of several key residues in the active pocket, which contributes to an understanding of the binding site (Li et al., 2020). As each amino acid in a protein is simulated, its RMSF value is used to identify its mobility and flexibility (Benson and Daggett, 2008). RMSF with a higher value indicates a more flexible residue, whereas RMSF with a lower value indicates a more stable system (Sadr et al., 2021). In this study, the selected four compounds showed a promising RMSF value that indicates firm attachment to the target protein binding pocket. A lower rGyr value indicates higher compactness, while a larger rGyr value signifies the dissociation of the compounds from the protein. The selected four compounds showed lower rGyr values than the control (vandetanib), thus denoting higher compactness (Supplementary Figure S6). A larger SASA value suggests a less stable structure, whereas a lower value indicates a tightly contracted complex of water molecules and amino acid residues (Ahammad et al., 2021). Higher stability was found for the selected four compounds (Supplementary Figure S6). Both MolSA and PSA are other two crucial parameters for the drug discovery process: MolSA quantifies the surface area of a molecule, and PSA indicates the surface area occupied by polar groups in protein–ligand complexes. Lower MolSA and PSA values denote high structural stability. In this study, the selected four compounds showed lower MolSA values than controls, and three compounds showed lower PSA values than controls except for CID 95842900, indicating higher structural stability (Supplementary Figure S6). Protein–ligand contact analysis in MD simulation identifies the protein residues that are commonly implicated in the dynamic motions of protein–ligand complexes. It also quantifies the strength of the interactions between the protein and ligands based on the specific amino acids involved (Pokhrel et al., 2021). In our study, multiple common interacting residues were found in hits and control ligands, indicating that the selected compounds bind to the protein’s active site. Simulation snapshots in 3D and 2D structures (0 ns and after 150 ns) (Supplementary Figures S7, S8), ligand torsions, and ligand–protein contact (Supplementary Figure S9) also demonstrate that the selected four biological compounds have strong binding capabilities in the protein active cavities. However, computational approaches can only predict the efficiency of drug candidates in an artificial environment. Varying outcomes were corroborated by evaluating four compounds according to distinct dimensions. So, further model organism trials are required to establish our study. This study can motivate researchers to conduct further wet lab experiments for cancer research.

## 5 Conclusion

Signaling pathways involving pRET tyrosine kinase regulate cell growth, proliferation, survival, differentiation, and hunger, which are responsible for several types of cancers. Drugs targeting pRET may reduce cancer progression by inactivating tumor metastasis and eliminating cancer stem cells by affecting their activity. The screening of compounds that can ameliorate cancer development is the primary focus of this research. Consequently, the study aims to identify potential lead compounds that impede the protein’s activity, thereby hindering the development of cancer. In this study, high-throughput virtual screening, molecular docking, ADME/T, MM-GBSA, and MD simulations revealed four compounds (CID 95842900, CID 124958150, CID 137030374, and CID 110126793) that can inhibit the activity of pRET tyrosine kinase, leading to a potential anticancer drug. Nevertheless, *in vivo* and *in vitro* studies are required to confirm the compounds’ activity against cancer.

## Data Availability

The original contributions presented in the study are included in the article/Supplementary Material; further inquiries can be directed to the corresponding authors.
