# Supplementary material for: Cheminformatics-based identification of phosphorylated RET tyrosine kinase inhibitors for human cancer
Source: Front Chem. 2024 Jul 17;12:1407331. doi: 10.3389/fchem.2024.1407331 (PMC11289668; doi:10.3389/fchem.2024.1407331)
Supplement: Supplementary file 1 [file DataSheet1.PDF]

Supplementary Files (Manuscript ID: 1407331)

Cheminformatics-based identification of phosphorylated RET Tyrosine kinase inhibitors for human cancer

Md. Enamul Kabir Talukder[1,2], Md. Aktaruzzaman[1,3], Noimul Hasan Siddiquee[1,4], Sabrina Islam[5], Tanveer A. Wani[6], Hamad M Alkahtani[6], Seema Zargar[7], Md Obayed Raihan[8], Md. Mashiar Rahman[2, *], Sushil Pokhrel[9, *] Foysal Ahammad[10, *]

Table S1. A list of various interactions between the selected six compounds and the phosphorylated RET tyrosine kinase domain (PDB id: 2IVU) was found during the XP molecular docking.

Compounds CID	Compounds IUPAC Name	H-bonds	Hydrophobic bonds	Other bonds
CID 95842900	2-[(2S)-2-[6-(pyridin-2-ylamino)pyrazin-2-yl]pyrrolidin-1-yl]acetamide	ALA807, TYR809	LEU730, VAL738, ALA756, ILE788, VAL804, TYR806, ALA807, TYR809, PHE815, LEU881	GLY731, LYS740, GLU805, LYS808, GLY810, SER811, GLY814, ARG878, SER891
CID 137030374	4-[(2S)-4-[(5-fluoropyridin-2-yl)methyl]morpholin-2-yl]-2-methyl-1H-pyrimidin-6-one	ALA807	LEU730, VAL738, ALA756, VAL757, LEU779, ILE788, LEU802, VAL804, TYR806, ALA807, TYR809, LEU881	GLY731, LYS740, LYS758, GLU775, GLU805, LYS808, GLY810, SER811, ARG878, ASN879, SER891, ASP892
CID 124958150	5-[[(2S)-2-(4-methyl-1H-pyrazol-5-yl)pyrrolidin-1-yl]methyl]quinoline	ALA807, ASP892	LEU730, VAL738, ALA756, ILE788, VAL804, TYR806, ALA807, TYR809, LEU881	GLY731, GLU732, GLY733, GLY736, LYS737, LYS758, GLU805, LYS808, GLY810, SER811, ARG878, ASN879, SER891, ASP892
CID 110126793	2-(3,5-dimethyl-1H-pyrazol-4-yl)-1-[(4S,5S)-4-hydroxy-5-imidazol-1-	ALA807, ARG87, ASP892	LEU731, VAL738, ALA756, ILE788, VAL804, TYR806, ALA807, TYR809, LEU881	GLY731, GLU732, GLY733, GLU734, LYS758, GLU805, LYS808, GLY810, SER811, GLY814, ARG878, ASN879, SER891, ASP892

	ylazepan-1-yl]ethanone			
CID 16672169	N-(furan-2-ylmethyl)-4,6-dimethyl-1H-pyrazolo[3,4-b]pyridin-3-amine	ALA807	LEU730, VAL738, ALA756, VAL757, LEU779, ILE788, LEU802, ILE803, VAL804, TYR806, ALA807, TYR809, LEU881	LYS728, GLY731, LYS758, GLU775, GLU805, LYS808, GLY810, SER811, SER891, ASP892
CID 110091816	4-[5-(3-fluorophenyl)-1,2-thiazol-4-yl]pyridin-2-amine	ALA807, ASP892	LEU730, VAL738, ALA756, VAL757, ILE788, VAL804, TYR806, ALA807, TYR809, LEU881	GLY731, GLU732, LYS758, GLU805, GLY810, SER811, ARG878, ASN879, SER891, ASP892
CID 110090418	1-[5-[(3-fluorophenyl)methyl]pyridine-3-carbonyl]azetidine-3-carboxamide	LEU730, SER811	LEU730, VAL738, VAL739, ALA756, VAL757, LEU779, ILE788, LEU790, LEU802, ILE803, VAL804, TYR806, ALA807, TYR809, LEU881, PHE893	GLY731, LYS758, GLU775, GLU805, LYS808, GLY810, SER811, GLY814, SER891, ASP892
CID 110090645	4-[5-[(2-fluorophenyl)methyl]pyridine-3-carbonyl]piperazin-2-one	ALA807	LEU730, VAL738, ALA756, VAL757, PHE776, LEU779, ILE788, LEU790, LEU802, ILE803, VAL804, TYR806, ALA807, TYR809, LEU881, VAL882, PHE893	GLY731, LYS740, LYS758, GLU775, GLU805, LYS808, GLY810, SER811, ARG878, SER891, ASP892
CID 125006174	1-[(3R)-3-[[6-(1H-pyrazol-4-yl)pyrazin-2-yl]methyl]pyrrolidin-1-yl]ethanone	ALA807, ASP892	LEU730, VAL738, ALA756, VAL757, LEU772, LEU779, ILE788, LEU790, LEU802, ILE803, VAL804, TYR806, ALA807, TYR809, LEU881, PHE893	GLY731, LYS758, GLU775, GLU805, LYS808, GLY810, SER811, SER891, ASP892
CID 125024702	3-[(2S)-4-[(5-chloropyridin-2-yl)methyl]morpholin-2-yl]pyrazin-2-amine	ALA807	LEU730, PHE735, VAL738, ALA756, ILE788, VAL804, TYR806, ALA807, TYR809, LEU881	GLY731, GLY733, GLY736, LYS737, LYS758, GLU805, LYS808, GLY810, SER811, ARG878, ASN879, SER891, ASP892
Vandetanib	*N*-(4-bromo-2-fluorophenyl)-6-methoxy-7-[(1-methylpiperidin-4-yl)methoxy]quinazolin-4-amine	ALA807	LEU730, VAL738, ALA756, VAL757, LEU779, ILE788, LEU802, VAL804, TYR806, ALA807, TYR809, LEU881	LYS728, GLY731, LYS740, LYS758, GLU775, GLU805, LYS808, GLY810, SER811, SER891, ASP892

Table S2. The table depicts the molecular mechanics with generalized born and surface area solvation (MM-GBSA) values of the lead ten compounds along with control drugs Vandetanib.

Compounds CID	ΔG Bind	ΔG Bind Coulomb	ΔG Bind Covalent	ΔG Bind Hbond	ΔGbind Lipo	ΔG Bind Packing	ΔG Bind Solv GB	ΔG Bind vdW
CID 95842900	-41.98	-21.93	5.90	-2.33	-14.35	-0.31	20.61	-29.56
CID 137030374	-32.32	-15.62	5.88	-1.02	-12.44	-0.42	23.68	-32.38
CID 124958150	-52.89	32.59	1.03	-1.40	-18.38	-0.46	-27.76	-38.50
CID 110126793	-50.20	58.40	2.74	-2.30	-13.44	-0.29	-58.66	-36.64
CID 16672169	-41.58	-15.92	2.43	-0.87	-12.43	-0.80	20.09	-34.07
CID 110091816	-44.60	-12.17	1.72	-2.12	-19.74	-0.37	18.54	-30.46
CID 110090418	-43.55	-13.04	4.37	-1.41	-20.17	-0.17	25.79	-38.91
CID 110090645	-39.50	-8.02	6.14	-0.57	-20.24	0	21.41	-38.22
CID 125006174	-40.19	-4.22	4.22	-0.72	-19.50	-0.31	20.53	-40.17
CID 125024702	-51.98	-15.16	1.69	-1.29	-16.55	-0.21	21.07	-41.50
Vandetanib	-29.64	14.79	-0.46	-9.89	-1.38	3.65	-36.35	-29.64

Table S3. The following is a compilation of pharmacokinetic properties for four selected compounds, encompassing physicochemical properties, lipophilicity, water solubility, pharmacokinetics, drug-likeness, medicinal chemistry, mutagenicity, and toxicity. The mutagenicity and toxicity aspects include evaluations of blood-brain barrier (BBB) permeability, hepatotoxicity (HT), carcinogenicity (CG), immunogenicity (IG), mutagenicity (MG), cytotoxicity (CT), and predicted toxicity class (PTC).

CID Number		CID-95842900	CID-137030374	CID-124958150	CID-110126793	CID-3081361 (control)
Physico-chemical Properties	MW (g/mol)	298.34	304.32	292.38	317.39	475.35
	Heavy atoms	22	22	22	23	30
	Arom. heavy atoms	12	12	15	10	16
	Rotatable bonds	5	3	3	4	6
	H-bond acceptors	5	6	3	4	6
	H-bond donors	2	1	1	2	1
Lipophilicity	Log Po/w(MLOGP)	-0.22	0.44	2.20	-0.02	3.72
Water Solubility	Log S (ESOL)	-1.93	-1.52	-3.69	-1.89	-5.89
Pharmacokinetics	GI absorption	High	High	High	High	High
Drug likeness	Lipinski, Violation	Yes; 0 violation	Yes; 0 violation	Yes; 0 violation	Yes; 0 violation	Yes; 0 violation
Medi. Chemistry	Synth. accessibility	3.43	3.31	2.90	3.54	3.28
Mutagenicity & Toxicity	BBB permeant	No	No	Yes	No	Yes
	HT (probability)	Inactive; 0.77	Inactive; 0.71	Inactive; 0.77	Inactive; 0.53	Inactive;0.51
	CG (probability)	Inactive; 0.56	Inactive;0.61	Inactive;0.51	Inactive;0.51	Inactive;0.52
	IG (probability)	Inactive; 0.93	Inactive;0.99	Inactive;0.69	Inactive;0.92	Inactive;0.99
	MG (probability)	Inactive; 0.72	Inactive;0.68	Inactive;0.53	Inactive;0.60	Inactive;0.57

	CT (probability)	Inactive; 0.87	Inactive;0.66	Inactive;0.53	Inactive;0.65	Inactive;0.75
	Predicted LD50	135mg/kg	400mg/kg	125mg/kg	500mg/kg	1000mg/kg
	PTC	3	4	3	4	4

Table S4. List of pharmacokinetics properties of selected last six-hit compounds, including physicochemical properties, lipophilicity, water solubility, pharmacokinetics, drug-likeness, medicinal chemistry, mutagenicity & toxicity (BBB, blood-brain barrier, HT, hepatotoxicity, CG, carcinogenicity, IG, immunogenicity, MG, mutagenicity, CT, cytotoxicity, and PTC, predicted toxicity class).

CID Number		CID 16672169	CID 110091816	CID 110090418	CID 110090645	CID 125006174	CID 125024702
Physico-chemical Properties	MW (g/mol)	242.28 g/mol	271.31 g/mol	313.33 g/mol	313.33 g/mol	271.32 g/mol	305.76 g/mol
	Heavy atoms	18	19	23	23	20	21
	Arom. heavy atoms	14	17	12	12	11	12
	Rotatable bonds	3	2	5	4	4	3
	H-bond acceptors	3	3	4	4	4	5
	H-bond donors	2	1	1	1	1	1
Lipophilicity	Log Po/w(MLOGP)	1.51	2.02	1.52	1.52	-0.17	-0.15
Water Solubility	Log S (ESOL)	-3.29	-3.92	-2.46	-2.82	-1.61	-1.99
Pharmacokinetics	GI absorption	High	High	High	High	High	High

Drug likeness	Lipinski, Violation	Yes; 0 violation	Yes; 0 violation	Yes; 0 violation	Yes; 0 violation	Yes; 0 violation	Yes; 0 violation
Medi. Chemistry	Synth. accessibility	2.55	2.59	2.35	2.34	3.18	3.10
Mutagenicity & Toxicity	BBB permeant	Yes	No	No	Yes	No	No
	HT (probability)	Inactive;0.66	Active; 0.66	Inactive;0.66	Inactive;0.85	Inactive;0.66	Inactive;0.64
	CG (probability)	Active; 0.54	Active; 0.53	Inactive;0.66	Inactive;0.71	Active; 0.61	Inactive;0.56
	IG (probability)	Inactive;0.91	Inactive;0.92	Inactive;0.66	Inactive;0.99	Inactive;0.90	Inactive;0.99
	MG (probability)	Inactive;0.51	Active; 0.63	Inactive;0.66	Inactive;0.76	Inactive;0.61	Inactive;0.70
	CT (probability)	Inactive;0.67	Inactive;0.75	Inactive;0.66	Inactive;0.77	Inactive;0.63	Inactive;0.67
	Predicted LD50	1600mg/kg	2000mg/kg	1300mg/kg	1000mg/kg	220mg/kg	800mg/kg
	PTC	4	4	4	4	3	4

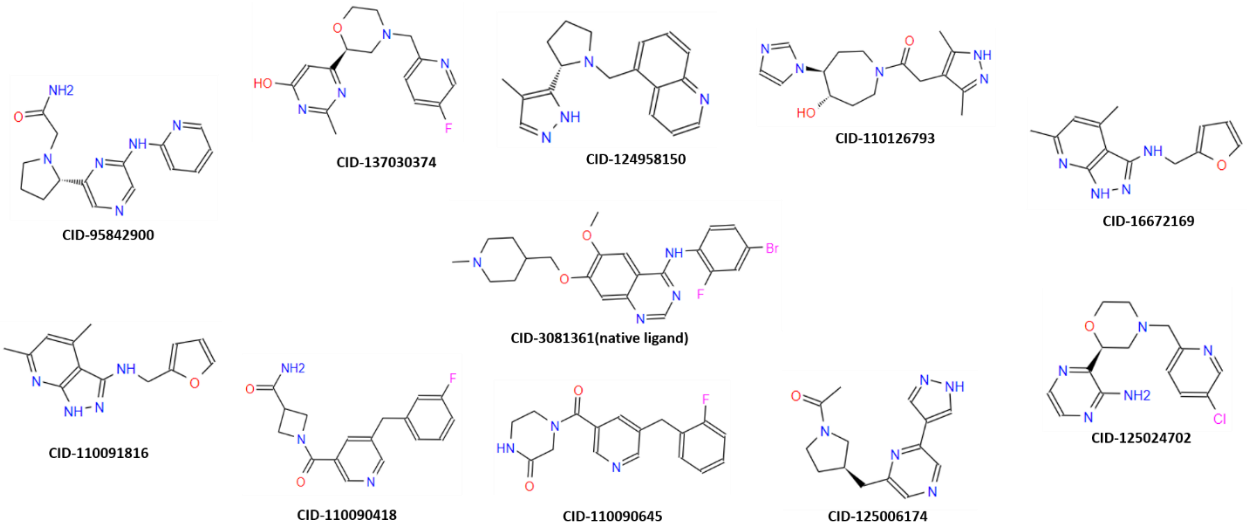

Figure S1. Two-dimensional (2D) structures of pRET tyrosine kinase domain (PDB id: 2IVU) inhibitor ZD6474 (at the center) and ten potential hit molecules are placed around the native ligands.

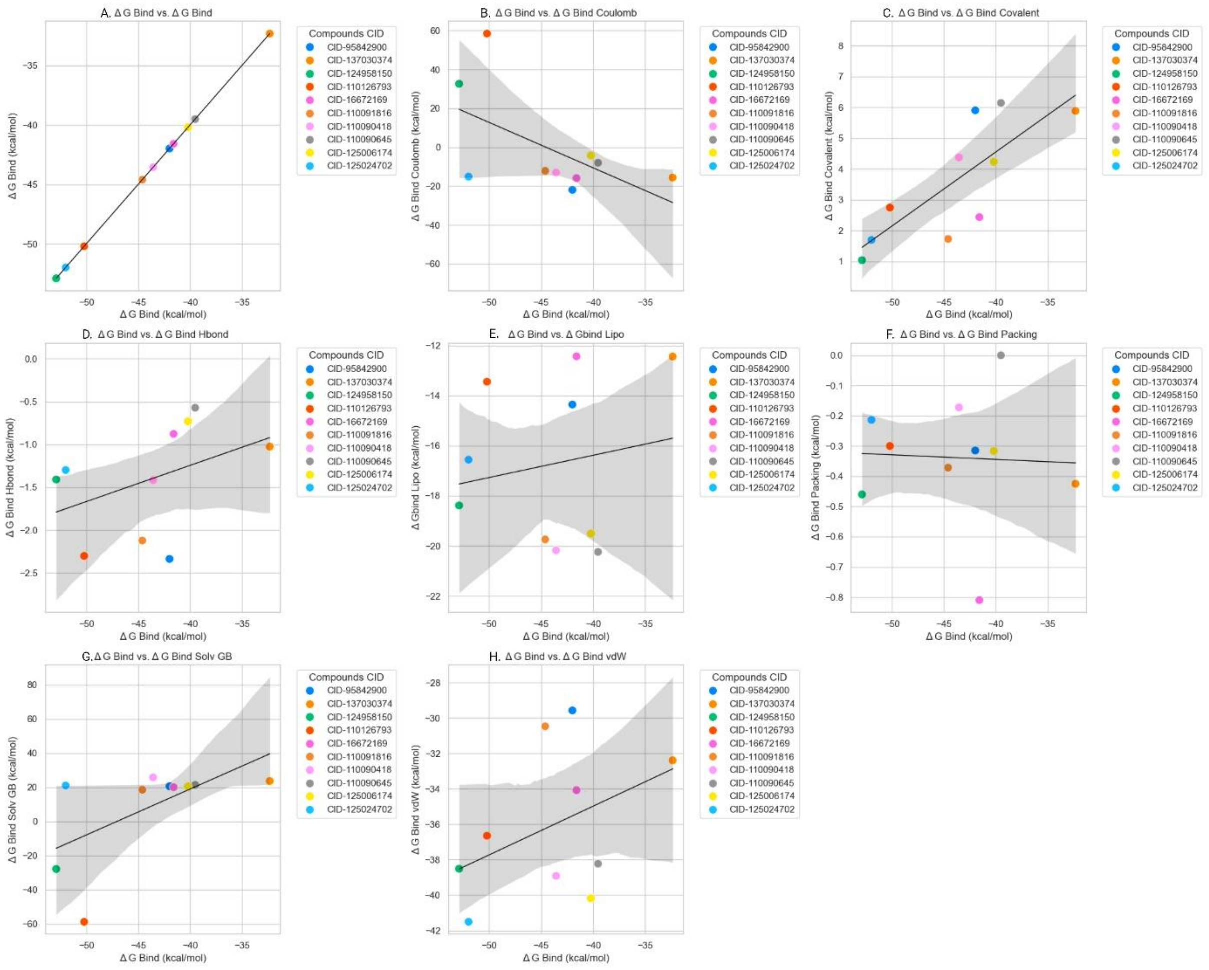

Figure S2. The figure shows scatter plots depicting the relationship between Δ G Bind (binding energy) and various binding energies for selected ten compounds. Each panel corresponds to a specific type of binding energy and shows its correlation with Δ G Bind for the chosen compounds, which are distinguished by different colors. The black line in each panel represents the linear regression fit for the data points. Plot A: Δ G Bind vs. Δ G Bind Coulomb Plot B: Δ G Bind vs. Δ G Bind Covalent plot C: Δ G Bind vs. Δ G Bind Hbond Plot D: Δ G Bind vs. Δ Gbind Lipo Plot E: Δ G Bind vs. Δ G Bind Packing Plot F: Δ G Bind vs. Δ G Bind Solv GB Panel G: Δ G Bind vs. Δ G Bind vdW This visualization aids in understanding the relationship between Δ G Bind and various binding energies for the compounds under study.

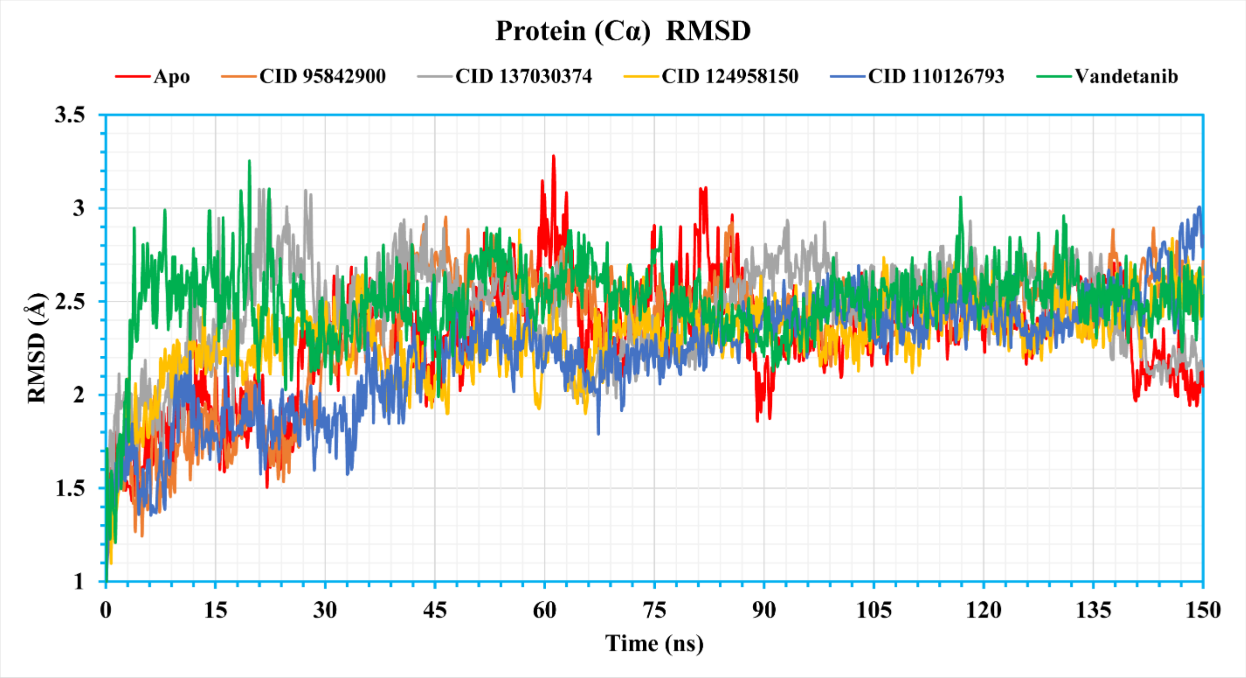

Figure S3. This figure depicts the RMSD values of protein carbon (Cα) atoms of phosphorylated RET kinase. Here, CID 95842900 (orange), CID 137030374 (grey), CID 124958150 (yellow), and CID 110126793 (blue) in comparison with Apo (red) and Control Vandetanib (green), respectively.

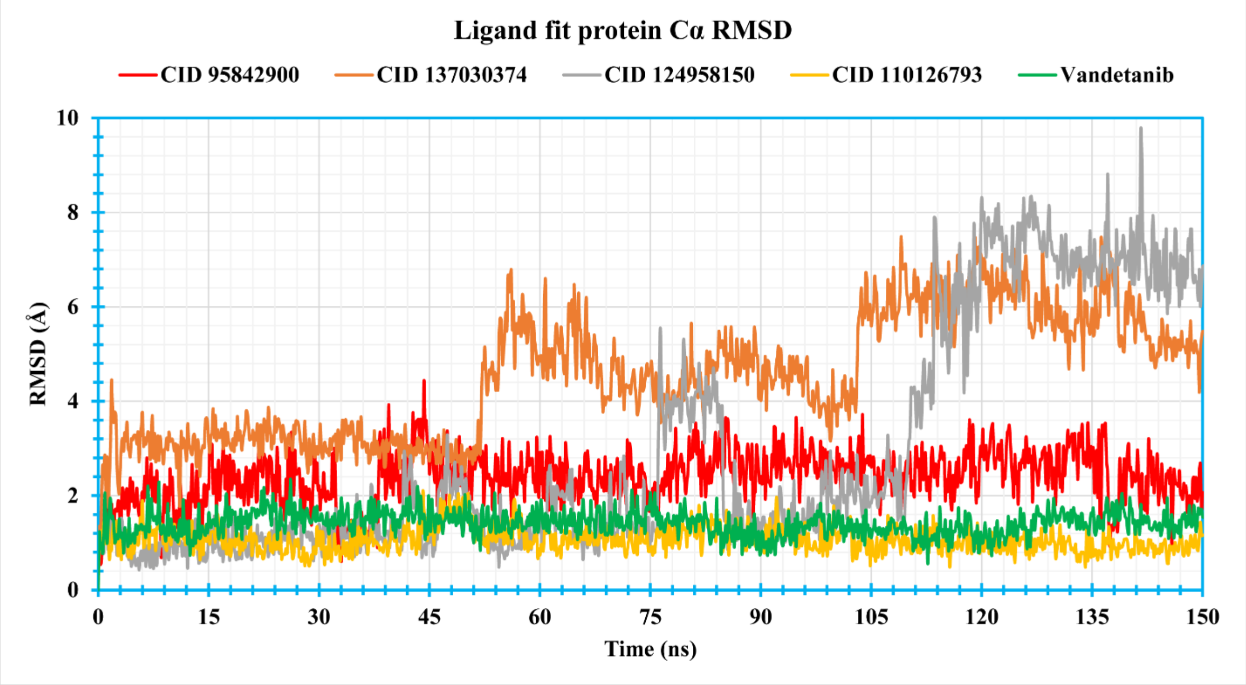

Figure S4. This figure illustrates the ligand RMSD values, indicated as CID 95842900 (red), CID 137030374 (orange), CID 124958150 (grey), CID 110126793 (yellow), and the control drugs Vandetanib (green), respectively.

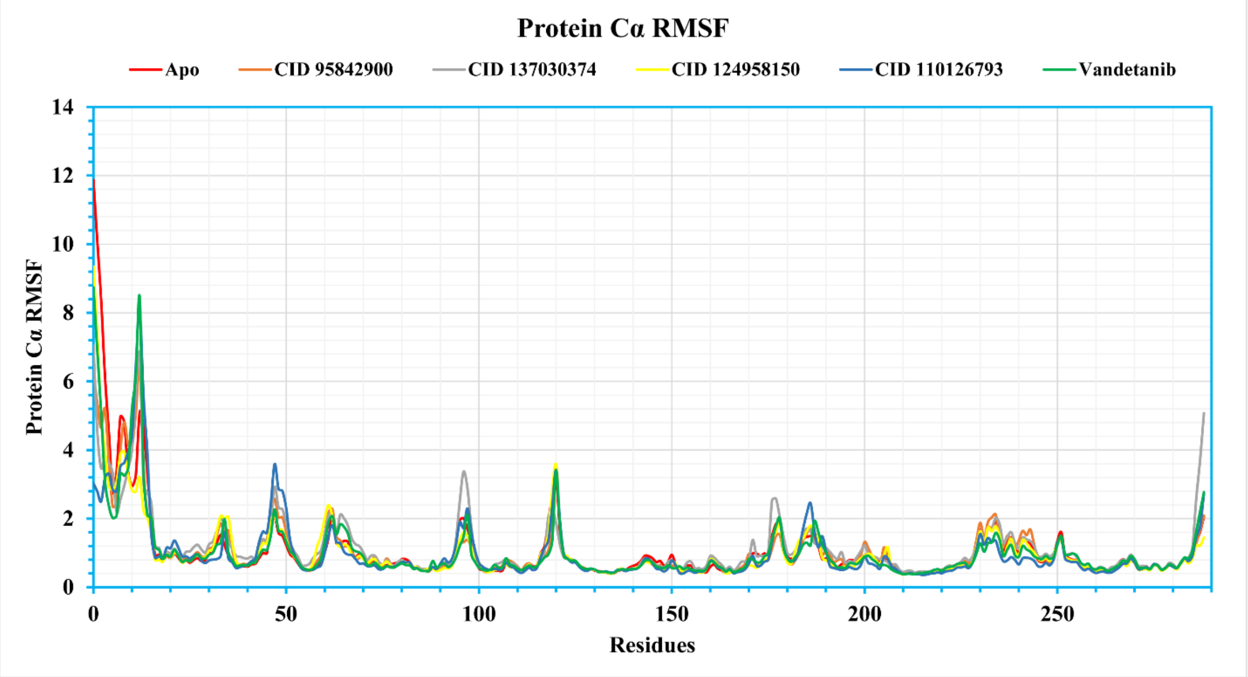

Figure S5. Showing the RMSF values of pRET tyrosine kinase were reclaimed from protein Cα atoms of the protein-ligand docked complexes. The RMSF of the CID 95842900 (orange), CID 137030374 (grey), CID 124958150 (yellow), and CID 110126793 (blue) are compared with two reference structures, Apo (red), and the Vandetanib (green) from the MD simulation.

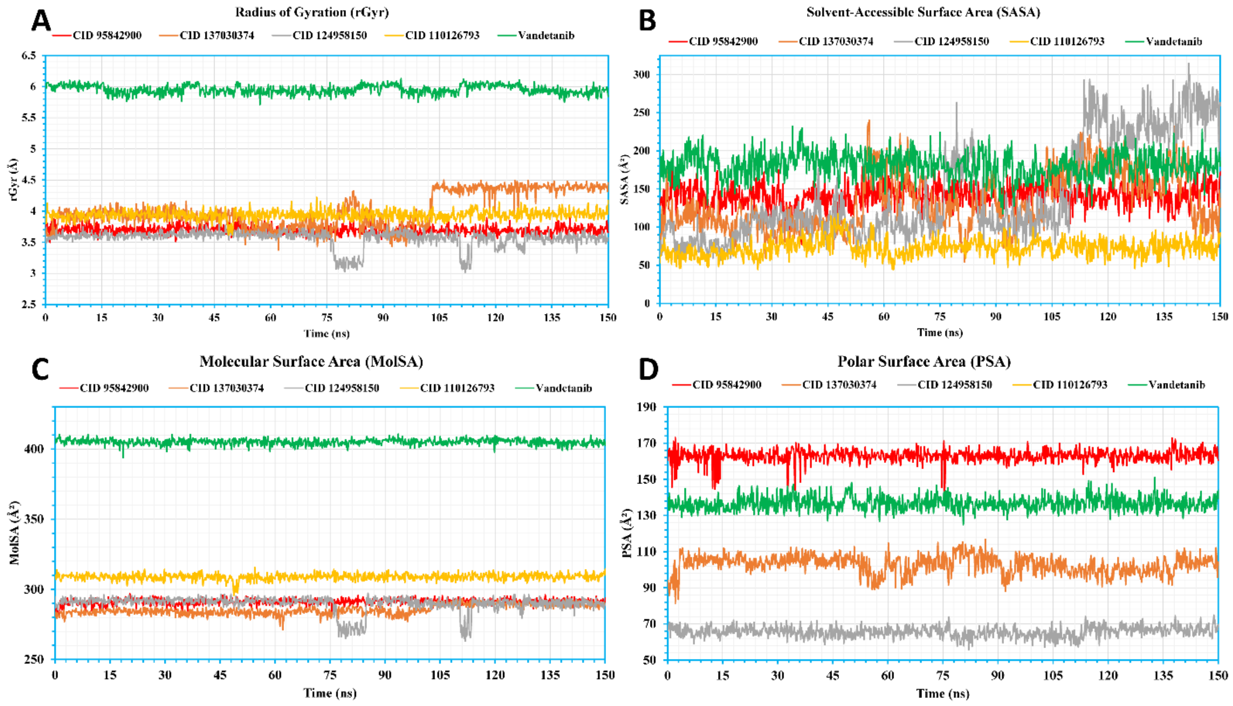

Figure S6. The graphs represent (A) radius of gyration (rGyr), (B) solvent-accessible surface area (SASA), (C) molecular surface area (MolSA), (D) polar surface area (PSA) values extruded from the complex structures of ligand atoms. All values were represented by CID 95842900 (red), CID 137030374 (orange), CID 124958150 (grey), CID 110126793 (yellow), and Vandetanib (reference) (green).

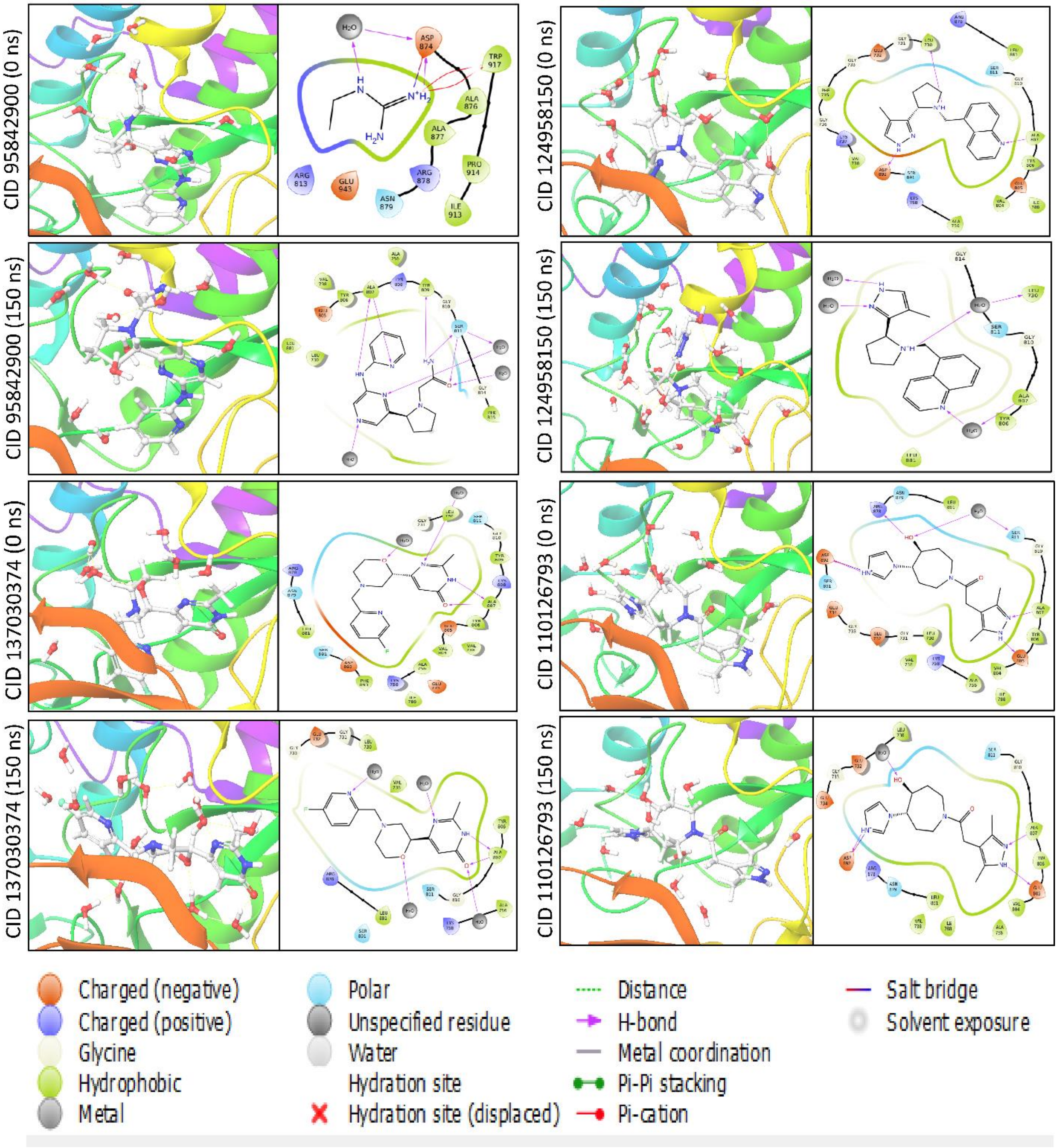

Figure S7. The molecular interactions from the molecular dynamics simulation obtained from 0ns and after 150 ns were reported for four lead compounds. Interactions obtained from simulation (0 ns) and after 150 ns (representative pose from the final snapshot of 150 ns) were compared to visualize hits demonstrating similar interactions and no loss of interactions.

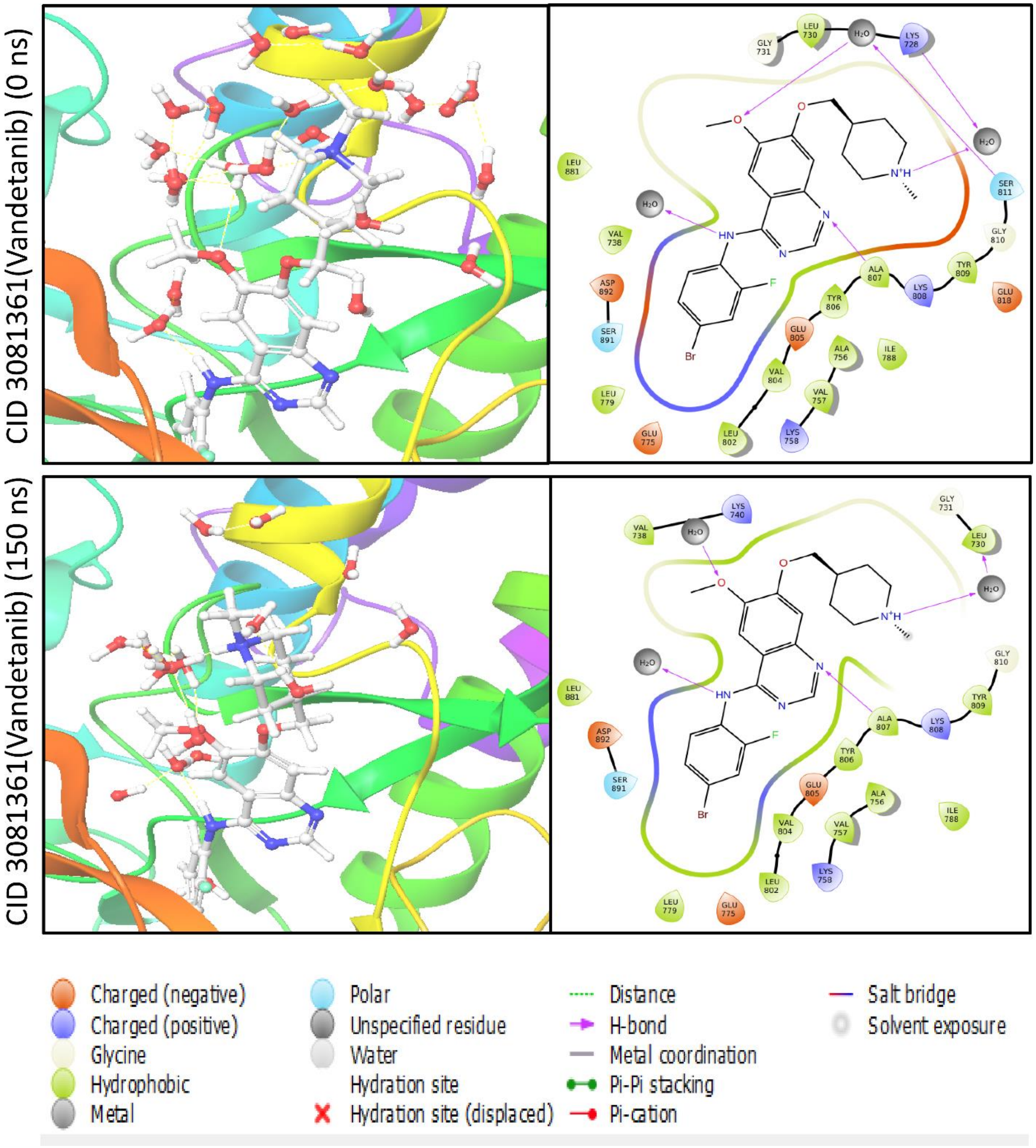

Figure S8. The molecular interactions from the molecular dynamics simulation obtained from 0ns and after 150 ns were reported for reference drugs (Vandetanib) in 3D and 2D structures.

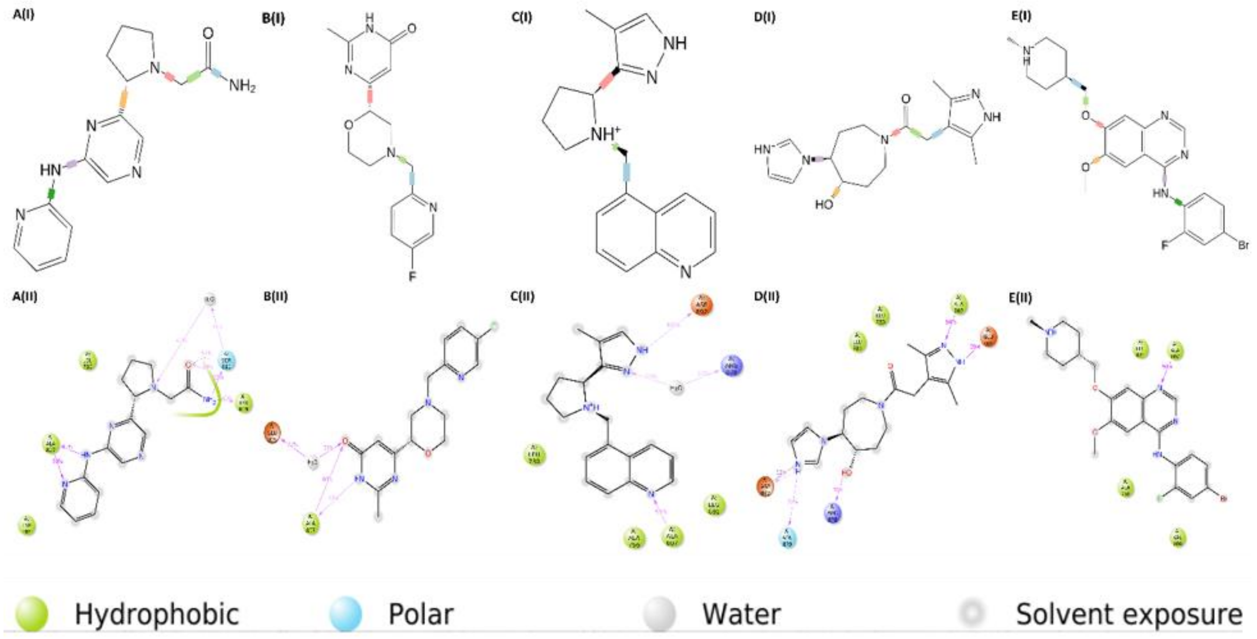

Figure S9. This figure exhibits the information about the ligand-torsions (above) and ligand-protein contacts (below), after 150 ns simulation. In this place, (A) (I) (II) CID 95842900, (B) (I) (II) CID 137030374, (C) (I) (II) CID 124958150, and (D) (I) (II) CID 110126793 and (E) (I) (II) CID 3081361 (control) respectively showing ligand-torsions & ligand-protein contacts.
